# Shared effects of *DISC1* disruption and elevated WNT signaling in human cerebral organoids

**DOI:** 10.1038/s41398-018-0122-x

**Published:** 2018-04-12

**Authors:** Priya Srikanth, Valentina N. Lagomarsino, Christina R. Muratore, Steven C. Ryu, Amy He, Walter M. Taylor, Constance Zhou, Marlise Arellano, Tracy L. Young-Pearse

**Affiliations:** Ann Romney Center for Neurologic Diseases, Brigham and Women’s Hospital and Harvard Medical School, Boston, MA USA

## Abstract

The development of three-dimensional culture methods has allowed for the study of developing cortical morphology in human cells. This provides a new tool to study the neurodevelopmental consequences of disease-associated mutations. Here, we study the effects of isogenic *DISC1* mutation in cerebral organoids. *DISC1* has been implicated in psychiatric disease based on genetic studies, including its interruption by a balanced translocation that increases the risk of major mental illness. Isogenic wild-type and *DISC1*-disrupted human-induced pluripotent stem cells were used to generate cerebral organoids, which were then examined for morphology and gene expression. We show that *DISC1*-mutant cerebral organoids display disorganized structural morphology and impaired proliferation, which is phenocopied by WNT agonism and rescued by WNT antagonism. Furthermore, there are many shared changes in gene expression with *DISC1* disruption and WNT agonism, including in neural progenitor and cell fate markers, regulators of neuronal migration, and interneuron markers. These shared gene expression changes suggest mechanisms for the observed morphologic dysregulation with *DISC1* disruption and points to new avenues for future studies. The shared changes in three-dimensional cerebral organoid morphology and gene expression with *DISC1* interruption and WNT agonism further strengthens the link between *DISC1* mutation, abnormalities in WNT signaling, and neuropsychiatric disease.

## Introduction

Modeling human neurodevelopment is a challenging but essential undertaking for addressing the cellular and molecular mechanisms underlying certain psychiatric diseases. Recent progress in human stem cell technologies allow us to examine developmental processes in human neural cells in a well-controlled system^1^. Induced pluripotent stem cells (iPSCs) have been derived from patients suffering from a variety of psychiatric diseases, and advances in genome engineering allow for efficient introduction and correction of genetic lesions that increase risk for disease. A number of differentiation protocols exist for directing stem cells to neuronal and astrocyte fates found along the neuraxis, with both monolayer and three-dimensional culture models available. These complementary methods have the potential to provide insights into the effects of gene disruption on neurodevelopmental processes.

Numerous laboratories have optimized monolayer differentiation protocols that efficiently generate neuronal cultures of a variety of fates. The use of these protocols is highly valuable and often essential if consistent and robust phenotypes are to be identified. However, a drawback of these cultures is that certain neurodevelopmental processes can only be examined in a three-dimensional (3D) environment more similar to in vivo development. To study human neurodevelopmental processes in a 3D structure, protocols have been developed to create cerebral organoids from embryonic stem cells and iPSCs^2–6^. Human stem cells are grown in aggregates, directed to a neural lineage via changes in media composition, and agitated in a spinning bioreactor to allow continued growth of organoids in 3D. Aggregates grow and develop over time, and can be cultured long term for greater than a year. These organoid cultures recapitulate aspects of neurodevelopment that cannot be examined in monolayer cultures. Such protocols are particularly valuable for studying windows of early cortical development such as the formation of certain progenitor zones and the migration of postmitotic precursor cells to recapitulate the inside–out development of cortical layers. Although protocols often result in a high level of variability between organoids, it is possible to identify morphological and regional differences between organoids when a strong genetic and/or pharmacological perturbation is introduced.

We previously have described the generation and characterization of an isogenic hiPSC model with disruption of the gene *DISC1* (Disrupted-in-Schizophrenia 1), a gene linked to elevated risk of mental illness^7^. *DISC1* was identified as a gene disrupted by a balanced translocation that increased risk for mental illness in a large Scottish family^8^. In our study, we showed that disruption of the gene at the site of the balanced translocation resulted in a loss of long *DISC1* isoforms, a reduction in the production of TBR2+ neural progenitor cells, and an elevation of baseline WNT signaling^7^. Early steps of the differentiation protocol employed in that study included the formation of 3D embryoid aggregates, but all data analyses were performed at later stages when cells were plated under monolayer conditions. Here, to better examine the morphological and molecular consequences of *DISC1* disruption in a 3D structure, we generated and analyzed cerebral organoids from isogenic hiPSCs with and without *DISC1* mutation.

We show that cerebral organoids with *DISC1* disruption are morphologically distinct from wild-type organoids. A few weeks after formation, wild-type cerebral organoids display well-defined rosette and ventricle-like structures that contain mitotic neural progenitor cells (NPCs) expressing PAX6 and TBR2. In contrast, cerebral organoids from *DISC1*-disrupted cells display smaller, disorganized rosette structures. Interestingly, wild-type organoids treated with a WNT agonist appear morphologically similar to *DISC1*-disrupted organoids, and WNT antagonism rescues the phenotype of *DISC1-*mutant organoids. *DISC1*-mutant organoids show a reduction in NPC proliferation, which is rescued by treatment with a WNT antagonist and phenocopied with a WNT agonist. Gene expression analyses in organoids show alterations in expression of genes critical to neuronal development, *POU3F2/BRN2* and *CALB1*. Further, gene expression analyses reveal that similiar gene expression changes are induced by *DISC1* mutation and transient treatment of wild-type cells with a WNT agonist. Taken together, these results support the hypothesis that loss of long *DISC1* isoforms results in an elevation of baseline WNT signaling in NPCs, resulting in morphological and neurodevelopmental consequences which may include altered cell fate and progenitor migration.

## Materials and methods

### Cerebral organoid culture

Cerebral organoids were generated largely as described^6^. IPSCs were dissociated and 3 × 10^6^ cells plated per well in an AggreWell 800 (Stemcell Technologies) centrifuged at 100×*g* for 3 min in iPSC media with low FGF2, changed to N2 supplement-containing neural induction media and transferred to 24 well plates on day 6. At this point, differentiation rounds were excluded if there was excessive debris present, or if the aggregates failed to form into cohesive organoids. At day 10, organoids were embedded in Matrigel droplets and transferred to N2/B27 supplement-containing media, and then cultured in organoid differentiation media in non-adhesive, 60 mm dishes thereafter. When indicated, cells were treated with 2 μM XAV939 (Stemgent), 3 μM CHIR99021 (Tocris Bioscience), or vehicle (DMSO) during days 6–19 of differentiation.

All cell lines were confirmed to be mycoplasma-free and to have the correct identity using STR profiling (Genetica) both prior to initiation of the study and at the conclusion of the study. Karyotyping was confirmed using the Nanostring Karytoping panel, and no gross chromosomal abnormalities were observed.

### Monolayer neuronal differentiation

Neuronal differentiation was performed using a previously described embryoid aggregate-based protocol^9^. Briefly, iPSC colonies were removed from mouse embryonic fibroblasts (MEFs) and cultured as embryoid aggregates in suspension for 4 days in iPSC media, followed by 2 days in N2 neural induction media. Day 7 aggregates were plated onto Matrigel-coated 6 well plates and maintained in N2 neural induction media, forming neuroepithelial structures. Cells were treated with 3 μM CHIR99021 or vehicle (DMSO) during days 7–17 of differentiation. At day 17, neural rosettes were enzymatically isolated using STEMDiff Neural Rosette Selection Reagent (Stemcell Technologies) and cultured in suspension for 7 days in N2/B27 neural induction media containing cAMP (1 μM, Sigma) and IGF-1 (10 ng/ml, Peprotech). Neural aggregates were dissociated using Accutase in the presence of 10 μM ROCK inhibitor and plated for final differentiation at ~85,000 cells/cm^2^ in neural differentiation media containing cAMP (10 μM, Sigma), IGF-1, BDNF, and GDNF (10 ng/ml, Peprotech). Cells were cultured for another 16 days to day 40.

### Immunostaining

Cells were fixed with 4% paraformaldehyde (Sigma), followed by membrane permeabilization and blocking with 0.2% Triton X-100 (Sigma) and 2% donkey serum (Jackson ImmunoResearch Laboratories). All quantifications were performed blinded to genotype in ImageJ. Antibodies: Nestin (R&D MAB1259), TBR2 (Abcam ab23345), PAX6 (Covance PRB-278P), BRN2 (Abcam Ab137469), PKCλ (BD Transduction Laboratory 610207), Cleaved caspase 3 (R&D Systems AF835), TUNEL (Sigma 12156792910), Acetylated alpha-tubulin (Cell Signaling 5335S), P73 (Abcam Ab40658), and Reelin (Abcam AB5364).

### EdU incorporation

Day 6 cerebral organoids were cultured in media containing 2 μM EdU for 2 days, followed by wash out and harvest on day 19. Cells were then fixed as described above, and EdU staining was carried out using the Click-iT EdU imaging kit (Invitrogen). Quantification of EdU+ was performed in a blinded manner: organoids were coded with random numbers such that the person performing the quantification did not know the identity of each sample until the conclusion of the analysis.

### Nanostring gene expression analysis

RNA was extracted from day 19 cerebral organoids using TRIzol (Life Technologies) or from day 40 neurons using the Pure Link RNA Mini Kit (Life Technologies), according to manufacturer’s instructions. Organoids were excluded from Nanostring or qPCR analyses if RNA yields were below 10 μg/μl. A custom 150-gene nCounter CodeSet was designed by NanoString Technologies. Data were analyzed with nSolver analysis software (Nanostring Technologies) and normalized to the total gene set. Assays were performed according to the manufacturer’s instructions.

### qPCR

RNA was reverse-transcribed, and cDNA was used for qPCR with Fast SYBR Green Master Mix (Life Technologies) on a ViiA 7 system (Life Technologies). Data were normalized to glyceraldehyde 3-phosphate dehydrogenase (GAPDH) expression using the ddCT method as described previously^10^. Primer sequences: GAPDH (forward, gggagccaaaagggtcatca; reverse, tggttcacacccatgacgaa), TBR2 (forward, gccatgcttagtgacaccg; reverse, ggactggaggtagtaccgc), Pax6 (forward, tctaatcgaagggccaaatg; reverse, tgtgagggctgtgtctgttc), CALB1 (forward, ggctccatttcgacgctga; reverse, gcccatactgatccacaaaagtt), POU3F2/BRN2 (forward, cggcggatcaaactgggattt; reverse, ttgcgctgcgatcttgtctat), Pancortin/OLMF1 domain B (forward, gctggtgggcctcaacacc; reverse ccgtgaacacatggtctgct); CALB2 (forward, actttgacgcagacggaaatg; reverse, gaagttctcttcggttcccag); NRG1 (forward, cggtgtccatgccttccat; reverse, gtgtcacgagaagtagaggtct), EAAT2 (forward, cctgacggtgtttggtgtcat; reverse, caagcggccactagccttag), FEZF2 (forward, actggccttttccatcgaga; reverse, tccgagtaactgagcagtgtc), VGLUT1 (forward, ctggggctacattgtcactca; reverse, gcaaagccgaaaactctgttg), VGAT (forward, acgtccgtgtccaacaagtc; reverse, aaagtcgaggtcgtcgcaatg).

### Data collection and statistics

All iPSC lines used in this study are isogenic apart from the DISC1 locus^7^. For each analysis, two wt lines and two DISC1 wt/μ lines were used. Numbers for gene expression analyses were chosen based upon initial pilot results examining the variability in gene expression across organoids. Data were analyzed using GraphPad PRISM 7 software. All quantified data in column graphs are expressed as mean ± SEM. Statistical significance was tested as indicated in the figure legends.

## Results

### Cerebral organoids express neural progenitor markers with ventricle-like, rosette, and dispersed morphologies

**Fig. 1:**
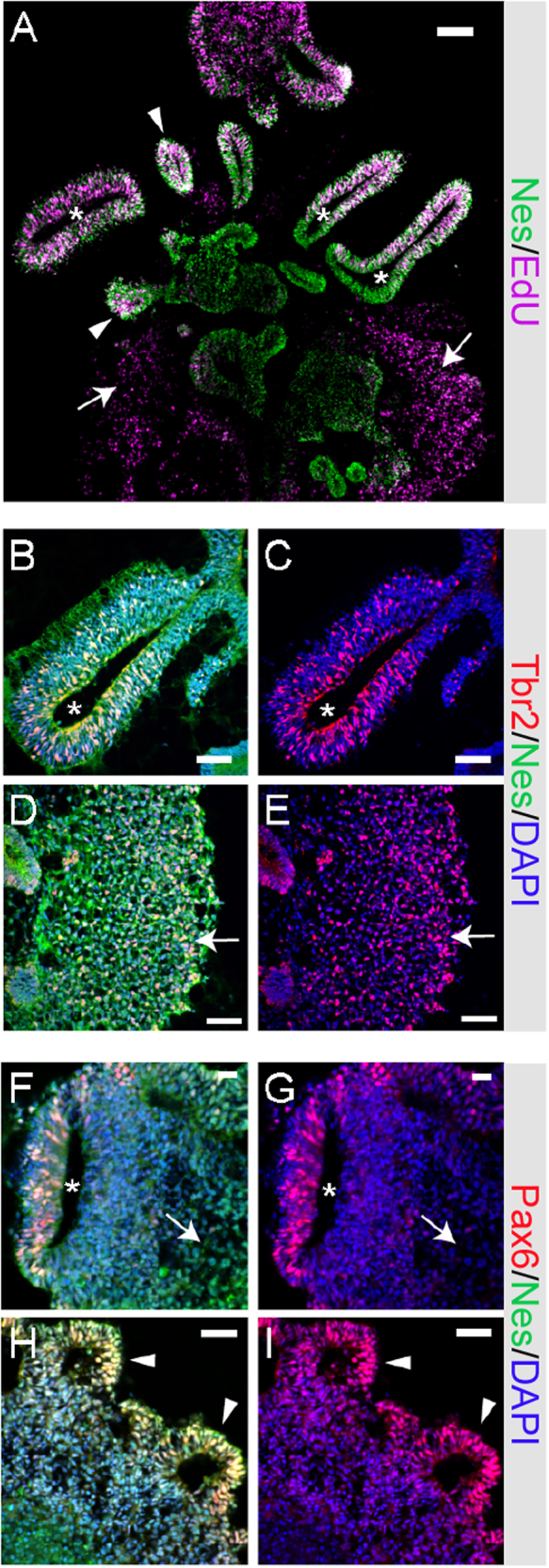
Cerebral organoids express neural progenitor markers with ventricle, rosette, and dispersed morphologies. **a** Organoids were differentiated to day 19 and pulsed with 2 μM EdU during days 6–7 before being fixed, sectioned, and stained for Nestin and EdU, showing areas of ventricle (asterisk), rosette (arrowhead), and dispersed (arrow) morphology. Day 19 organoids immunostained for **b**–**e** Tbr2 and Nestin, or **f**–**i** Pax6 and Nestin, showing areas of ventricle (asterisk), rosette (arrowhead), and dispersed (arrow) morphology. Scale bars: 100 μm (**a**), 50 μm (**b**–**e**, **j**–**l**), 20 μm (**f**–**i**)

To generate cerebral organoids, we used a protocol similar to that published by the Knoblich lab^6^ with the following exceptions: we dissociated iPSCs from MEFs using Accutase instead of dispase/trypsin to obtain a single cell suspension, and we used AggreWells for initial embryoid body (EB) formation (3 × 10^6^ cells per Aggrewell) instead of round-bottom plates. To characterize the cultures, organoids were pulsed with EdU during days 6 and 7 of differentiation, then continued in culture for 12 days without EdU. These aggregates were fixed, cryosectioned, and immunostained to examine the morphology of aggregates at this early stage of neurodevelopment. At day 19, wild-type organoids were composed of neural progenitor lineages that line fluid-filled cavities, similar to ventricles, along with regions of neural rosettes. There also were areas of “dispersed” cells that lacked discernible structure. Figure 1a shows a representative organoid stained for EdU and the NPC cytoskeletal marker Nestin (NES), with examples of each type of structural morphology. Interestingly, subsets of cells were found to express a marker of radial glia (PAX6) and a marker of intermediate progenitor cells (TBR2/EOMES) in areas with disparate morphologies including: cells lining the large ventricle-like structures, in rosettes, and in dispersed areas (Fig. 1b–i). This initial characterization revealed that organoids, at this time point, contain forebrain neural progenitors with heterogeneous structural organization.

### *DISC1* mutation disrupts cerebral organoid morphology in a manner that is phenocopied by WNT agonism

**Fig. 2:**
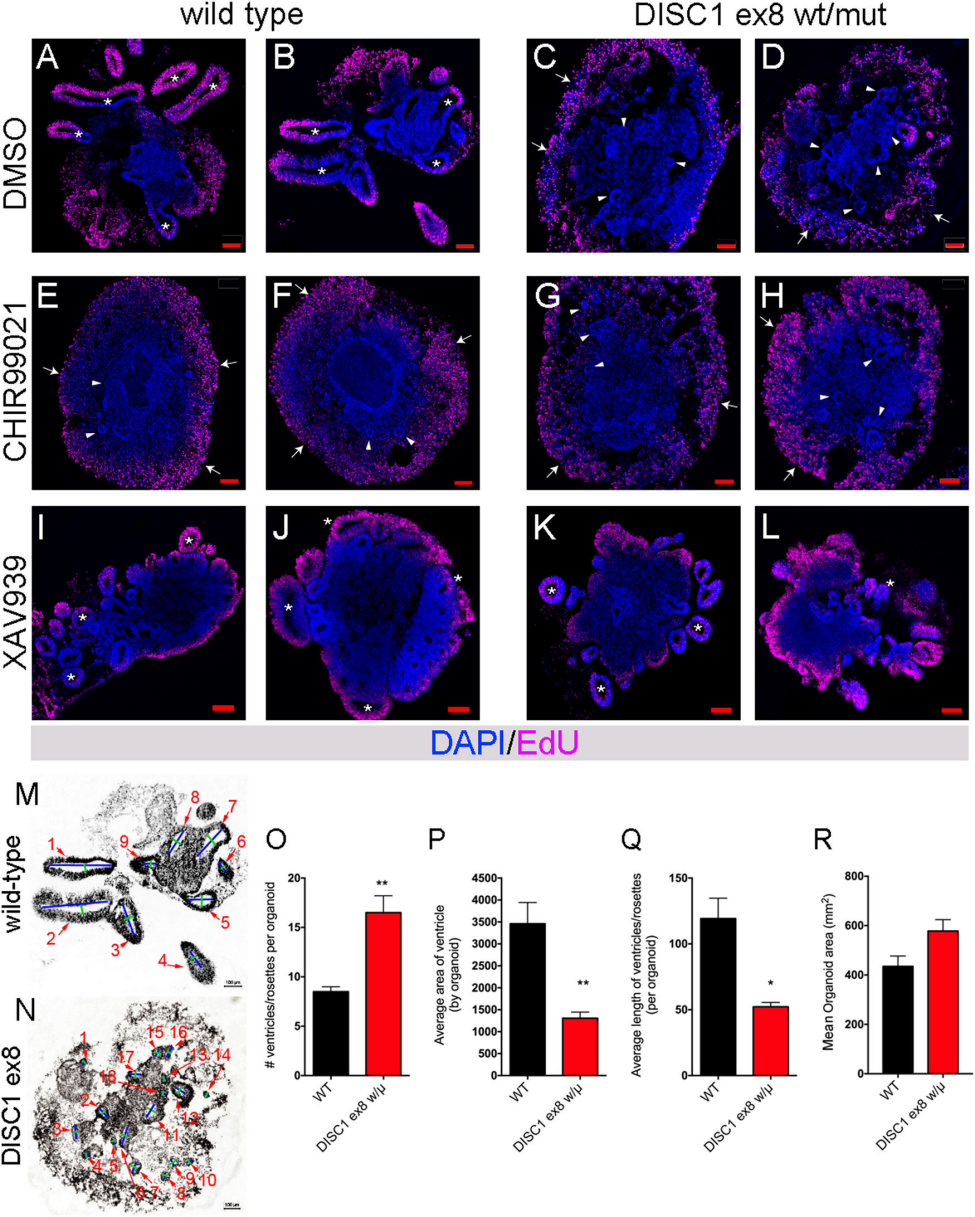
*DISC1* disruption alters cerebral organoid morphology. **a**–**l** Wild-type and *DISC1*-disrupted organoids were pulsed with EdU for 2 days (culture days 6–7), then fixed, sectioned, and stained for EdU at day 19. Two representative organoids are shown for wild-type (**a**, **b**, **e**, **f**, **i**, **j**) and *DISC1*-disrupted (**c**, **d**, **g**, **h**, **k**, **l**) lines treated with either DMSO (vehicle, **a**–**d**), WNT agonist CHIR99021 (**e**–**h**), or WNT antagonist XAV939 (**i**–**l**). Well-defined ventricles/rosettes are labeled with asterisks, small poorly defined rosettes labeled with arrowheads, and areas of dispersed morphology labeled with arrows. Scale bars = 100 μm. Example measurements of ventricles/rosettes (labeled with red arrows) with blue lines labeling length and green lines labeling width, are shown in **m** (same image as **b**) and **n** (same image as **d**). Morphology of DMSO-treated wild-type or *DISC1*-disrupted organoids was quantified, blinded, in sections from the middle of the organoid, by counting number of ventricles or rosettes visible per organoid (**o**), average ventricle/rosette area per organoid (**p**), average length of the long axis of ventricles/rosettes per organoid (**q**), and organoid area (**r**). Data were derived from four independent differentiations. *n* = 4 (wt), 4 (DISC1 wt/μ). Statistics: two-tailed Student’s *t-*test. *p < 0.05, **p < 0.01. Variances were not significantly different in **o**, **p**, and **r**. Variances were significantly different in **q**, and *t*-test was performed with Welch’s correction. See also Supplementary Figure 1 for normalization per total area for each organoid

We previously described the generation of isogenic hiPSC lines with *DISC1* disruption within exon 8^7^, very near the site of a balanced translocation linked to mental illness in a large Scottish family^8^. Using TALEN targeting of exon 8, multiple clonal lines were derived that were wild-type or that harbored a premature stop codon within exon 8. Following differentiation to neural progenitor and neuronal fates of the cerebral cortex, we found that long splice variants of *DISC1* were reduced by half in heterozygous lines, and eliminated in homozygous lines^7^. As the balanced translocation leads to elevated risk for mental illness in the heterozygous form (homozygous individuals have not been reported), we chose here to focus upon the model of *DISC1* disruption most similar to the disease state, heterozygous exon 8 disruption (*DISC1* ex8 wt/μ). To minimize the potential for clone and differentiation-specific effects, two clonal wild-type lines and two clonal *DISC1* ex8 wt/μ lines were paired and analyzed over more than 10 differentiations. Unexpectedly, organoids derived from *DISC1*-mutant iPSCs displayed aberrant morphology (Fig. 2c, d). Mutant organoids lacked large and well-defined ventricle-like structures, and contained more small, disorganized rosette structures, and more areas of dispersed morphology when compared to wild-type organoids (Fig. 2a, b). These morphological changes were quantified for three differentiation rounds by counting the number of ventricles or rosettes present per organoid, average area of ventricles/rosettes per organoid, average length of the long axis of ventricles/rosettes present in each organoid, and total organoid area (Fig. 2o–r). As there are no clear criteria for differentiating rosettes from ventricle-like structures given the 3D structure of organoids, rosettes and ventricle-like structures were pooled for analysis, with example length/width measurements shown in Fig. 2m–n. Quantification revealed significant alterations with DISC1 mutation consistent with visible morphological changes, with increased numbers of rosettes per organoid (Fig. 2o), decreased ventricle/rosette area (Fig. 2p), and decreased length of the long axis of ventricles/rosettes (Fig. 2q). Notably, there was no clear difference in organoid area between wild-type and DISC1-mutant organoids (Fig. 2r).

Multiple studies have previously linked *DISC1* function to WNT signaling^7,11,12^. In our initial report describing *DISC1*-mutant neural cells in monolayer culture, we found that *DISC1* disruption resulted in an elevation in baseline WNT signaling, which caused subtle alterations in cell fate at a neuronal time point (differentiation day 40)^7^. To examine whether elevated WNT signaling could induce the altered morphology observed in cerebral organoids with *DISC1* disruption, organoids were treated with a WNT agonist (GSK3β inhibitor CHIR99021, 3 μM^13^) during days 6–19 of culture. Interestingly, incubation with the WNT agonist resulted in a structural change in wild-type organoids that phenocopied the effects of *DISC1* disruption, including increased areas of dispersed cell morphology, and more small and disorganized rosette structures (Fig. 2e, f). WNT agonism of *DISC1*-mutant organoids did not have a marked effect on morphology (Fig. 2g, h). Furthermore, WNT antagonism (using tankyrase inhibitor XAV939, 2 μM^14^) similarly affected both wild-type and *DISC1* ex8 wt/μ organoids to produce small, well-defined rosette/ventricle structures (Fig. 2i–l). Quantification showed that WNT antagonism with XAV939 rescued the phenotype of increased ventricle/rosette numbers with *DISC1* disruption (normalized to organoid area, Supplemental Figure 1A).

### *DISC1* disruption reduces proliferation but does not affect apoptosis in early cerebral organoids

**Fig. 3:**
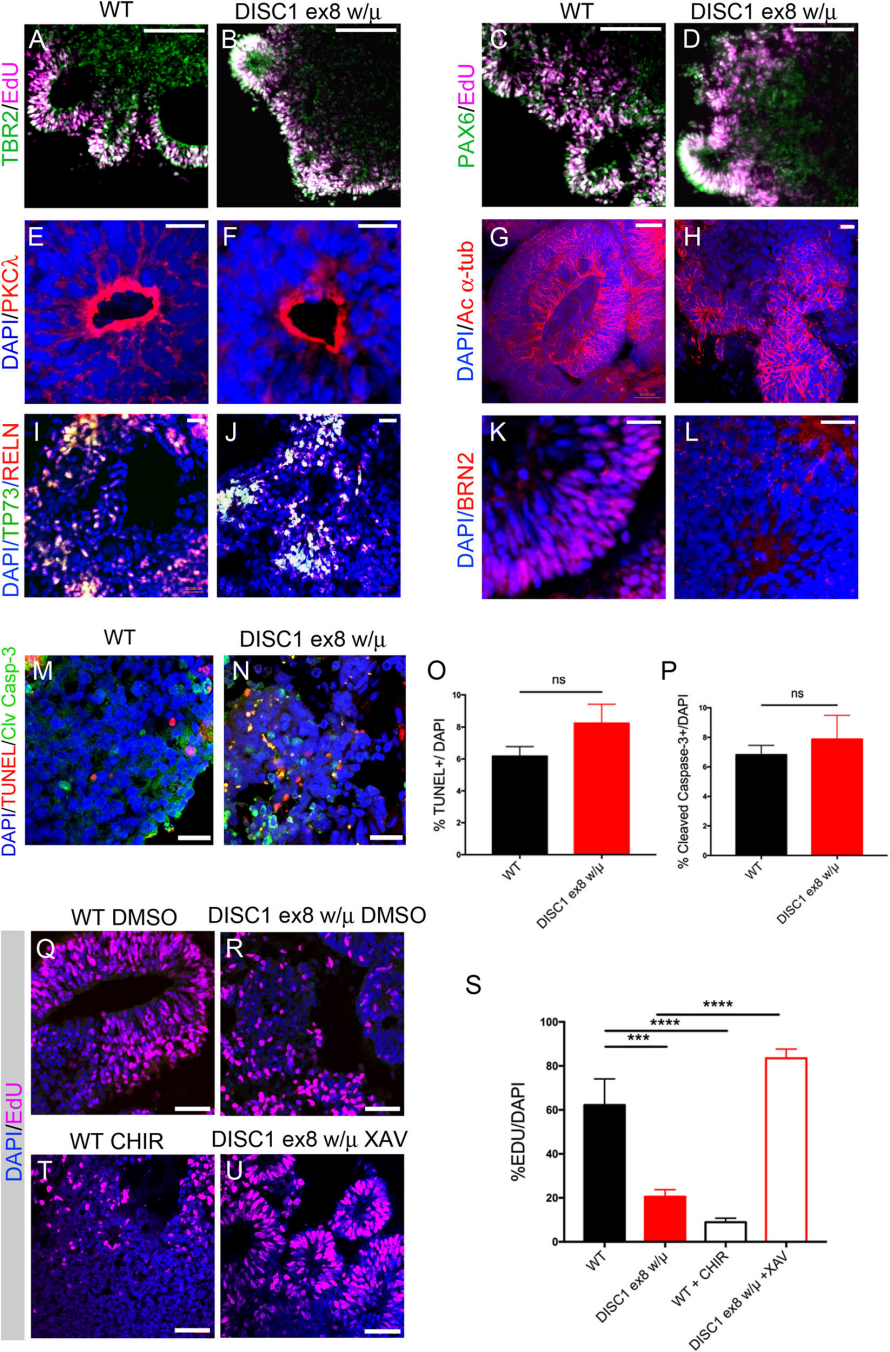
*DISC1*-mutant organoids exhibit decreased BRN2 expression and reduced proliferation that is rescued by WNT antagonism. Immunostaining was performed on WT and *DISC1*-mutant day 19 organoids for EdU incorporation and markers as shown. Expression of cell fate markers TBR2, PAX6, P73, Reelin and polarity markers PKC-λ, and acetylated-α-tubulin were grossly unchanged (**a**–**j**). However, immunostaining of BRN2 was markedly reduced in *DISC1*-mutant organoids (**k**, **l**). Scale bars: **a**–**d** 100 μm, **e**–**h** 20 μm, **i**–**j** 50 μm, **k**–**l** 20 μm. **m**, **n** WT and *DISC1* exon 8 wt/μ organoids were immunostained at day 19 for markers of apoptosis (TUNEL and Cleaved Caspase 3). **o**, **p** Quantification of percentage of DAPI positive nuclei positive for TUNEL or cleaved caspase-3 shows no difference with *DISC1* disruption. **q**–**u** WT and *DISC1*-mutant organoids with WNT agonism (CHIR) or WNT antagonism (XAV) were pulsed with EdU for 2 days (culture days 6–7), then fixed, sectioned, and stained for EdU and DAPI at day 19. One representative image for each condition is shown. **s** Percentage of EdU-positive nuclei were quantified. Data were derived from three independent differentiations. Statistics: **o**, **p** Variance was significantly different between conditions, Welch’s *t*-test. **s** Variance was significantly different between conditions, one-way ANOVA with Geiser–Greenhouse correction, Sidak’s multiple comparisons test. ***p < 0.001, ****p < 0.0001. Scale bars: **m**, **n**,** q**, **p**, **t**, **u**: 50 μm

We compared expression of certain cell fate markers in wild-type and DISC1 exon 8-mutant organoids. Subventricular zone (SVZ) intermediate progenitor marker TBR2 and dorsal neocortical and ventricular zone (VZ) progenitor marker PAX6^15^ were expressed similarly in WT and *DISC1*-disrupted organoids (Fig. 3a–d). The polarity of progenitors in neural rosettes was similar, with apical membranes directed interiorly, as identified by PKCλ and acetylated α-tubulin immunostaining (Fig. 3e–h). Markers of Cajal–Retzius cells, Reelin and p73, were identified in both WT and mutant organoids (Fig. 3i, j). Interestingly, expression of late neocortical progenitor and layer II–V neuronal marker BRN2/POU3F2, which has been shown to be critical for proper cortical lamination^16^, was markedly decreased with *DISC1* mutation (Fig. 3k–l). Expression of neural progenitor markers *PAX6* and *TBR2 (EOMES)*, glutamatergic neuronal marker *VGLUT1 (SLC17A7)* and GABAergic neuronal marker *VGAT (SLC32A1)* were not significantly changed in d19 DISC1-mutant organoids, as measured by qPCR (Supplemental Figure 2).

### *DISC1* mutation does not alter apoptosis but results in a WNT-dependent decrease in EdU incorporation

We next investigated whether perturbations in proliferation or apoptosis were associated with the described morphologic alterations in *DISC1*-mutant cerebral organoids. To evaluate whether altered apoptosis also contributed to aberrant morphology, day 19 organoids were used for TUNEL immunohistochemistry and immunostaining for activated cleaved Caspase 3. There was no significant difference in the percentage of TUNEL+ or cleaved Caspase-3+ cells with *DISC1* mutation, suggesting that altered apoptosis does not significantly contribute to the observed phenotype (Fig. 3m–p).

To examine potential changes in proliferation, cerebral organoids were pulsed with EdU during day 6–7 of differentiation, cultured in the presence of either DMSO, CHIR99021, or XAV939 during days 6–19, and then harvested and imaged at day 19 (Fig. 3q–u). As EdU was found to only incorporate into the outer areas of organoids, likely due to limited permeation of the 3D structure (Fig. 2a–l), EdU quantification was performed in these areas, defined in a blinded manner based on maximal penetration of EdU. Quantification showed that *DISC1* disruption, as well as WNT agonism with CHIR99021, resulted in a decreased percentage of EdU-positive cells, whereas treatment of *DISC1*-mutant organoids with WNT antagonist XAV939 rescued this EdU incorporation phenotype (Fig. 3s). This implicates increased WNT activity as a mechanism resulting in decreased proliferation in *DISC1*-mutant organoids at this early developmental time point.

### *DISC1* disruption alters expression of genes implicated in neurodevelopment and migration and is mimicked by WNT agonism

**Fig. 4:**
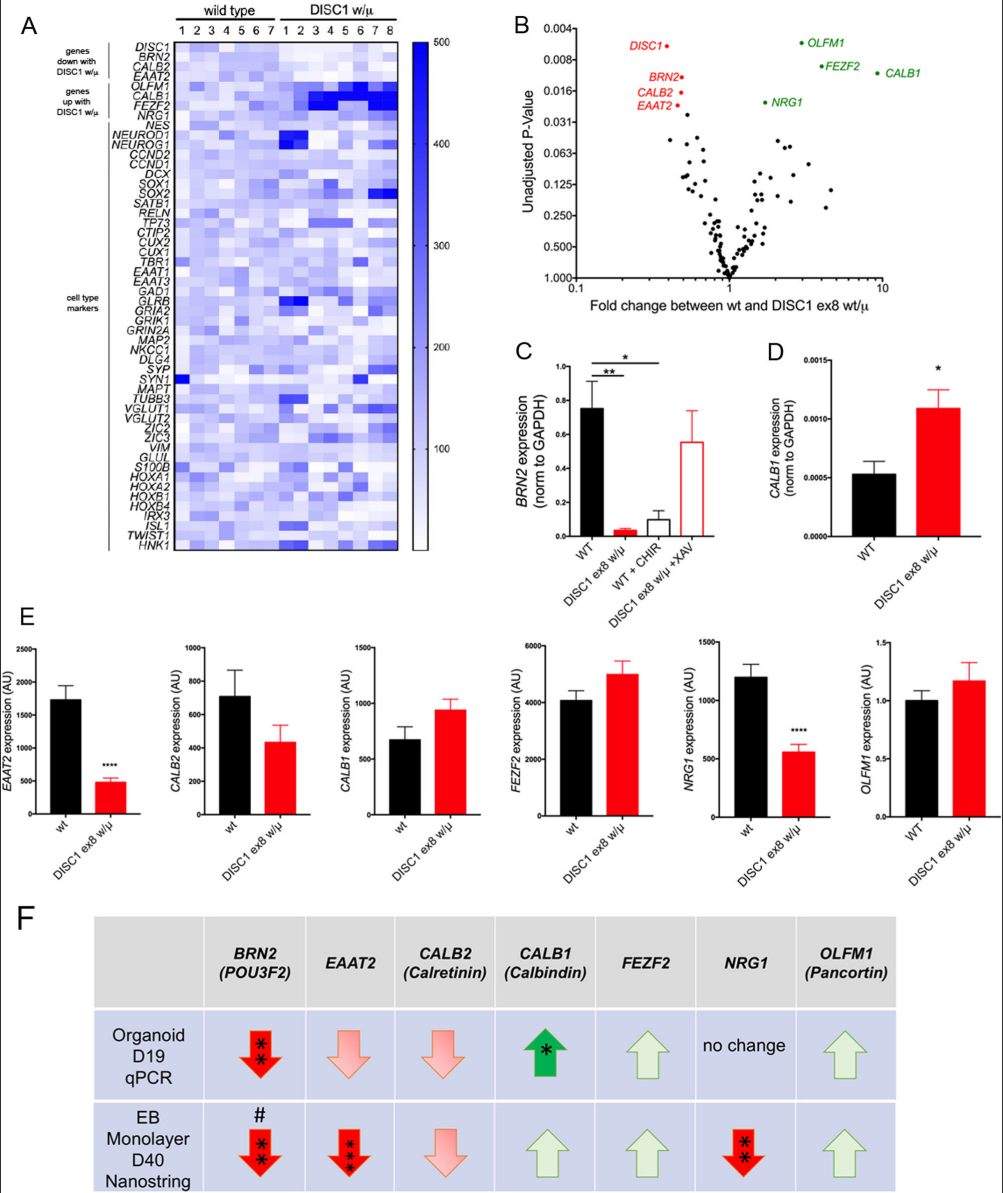
*DISC1* disruption alters *BRN2* levels in organoids and monolayer cultures. Wild-type and *DISC1* ex8 wt/μ organoids were harvested at day 19 and RNA was used for Nanostring analyses. **a** Heat map of cell type markers for wild type (wt) and DISC1 ex8 wt/μ organoids. **b** Volcano plot of gene expression changes in *DISC1* ex8 wt/μ vs. wild-type organoids are shown. Statistics: Student’s *t*-test, unadjusted *p*-values plotted; *n* = 7 for wild-type, 8 for *DISC1* wt/μ, from four independent differentiations. See also Supplementary Table 1. **c**, **d** qPCR was performed for *BRN2* and *CALB1* on RNA derived from day 19 organoids. Data were derived from three independent differentiations. For each differentiation, over five organoids were pooled for analysis. **e** Wild-type and *DISC1* ex8 w/μ iPSCs were differentiated to NPCs using an EB protocol^7^ and subsequently dissociated and plated as monolayer culture. Day 40 neuronal RNA was harvested and used for Nanostring. Data were derived from three to seven independent differentiations. Statistics: **c** One-way ANOVA with Sidak multiple comparisons test, **d,e** Student’s *t*-test, *p < 0.05, **p < 0.01, ****p < 0.0001. **f** Table summarizing gene expression changes across both differentiation methods: organoid day 19 qPCR (from **c**, **d** and other data not shown) and day 40 monolayer Nanostring data. Asterisks indicate significance by two-tailed Student’s *t*-test; ^#^indicates data published in ref. ^7^

To investigate factors contributing to the altered morphology of *DISC1*-mutant cerebral organoids, we assayed gene expression in day 19 organoids using a custom Nanostring panel of 150 genes related to neuronal development, maturity, and cell signaling. A heat map of expression of a subset of genes reveals the high organoid-to-organoid variability at the molecular level (Fig. 4a). Genes shown in the heat map mark particular subsets of cells in the central nervous system (CNS), revealing the variety in the cell types present in wild type and mutant organoids at day 19. These include markers of excitatory neurons (*GRIA2, GRIK1, GRIN2A, VGLUT1, VGLUT2)*, inhibitory neurons (*GAD1, GLRB*), caudally located neurons of the hindbrain and spinal cord (*HOXA1, HOXA2, HOXB1, HOXB4, IRX3, ISL1*) and neural crest cells (HNK1), as well as cortical layer-specific markers (*RELN, SATB1, CUX1, CTIP2, TP73, TBR1*).

Due in part to inherent variability of cerebral organoids at the molecular level, no genes achieved statistical significance with correction for multiple comparisons across the 150 genes examined. Using unadjusted *p*-values, *DISC1* expression was one of the more significant findings, which is consistent with our previous findings of decreased *DISC1* expression with this genomic mutation due to nonsense-mediated decay (as previously observed in neural cultures derived from these cells^7^). Among the other gene changes highlighted by this discovery set were decreased expression of *BRN2*/*POU3F2*, Calretinin (*CALB2*), and *EAAT2*/*SLC1A2*, and increased expression of Pancortin (*OLFM1*), Calbindin (*CALB1*), *FEZF2*, and *NRG1* (Fig. 4a, b).

To test the validity of observed gene expression changes, a set of replication experiments were performed to measure a selection of genes using qPCR. This confirmed decreased *BRN2* and increased *CALB1* expression (Fig. 4c, d). Although some other genes showed trends for altered expression in agreement with Nanostring results (decreased *EAAT2* and *CALB2*; increased *FEZF2* and *OLFM1*), these did not reach significance.

**Fig. 5:**
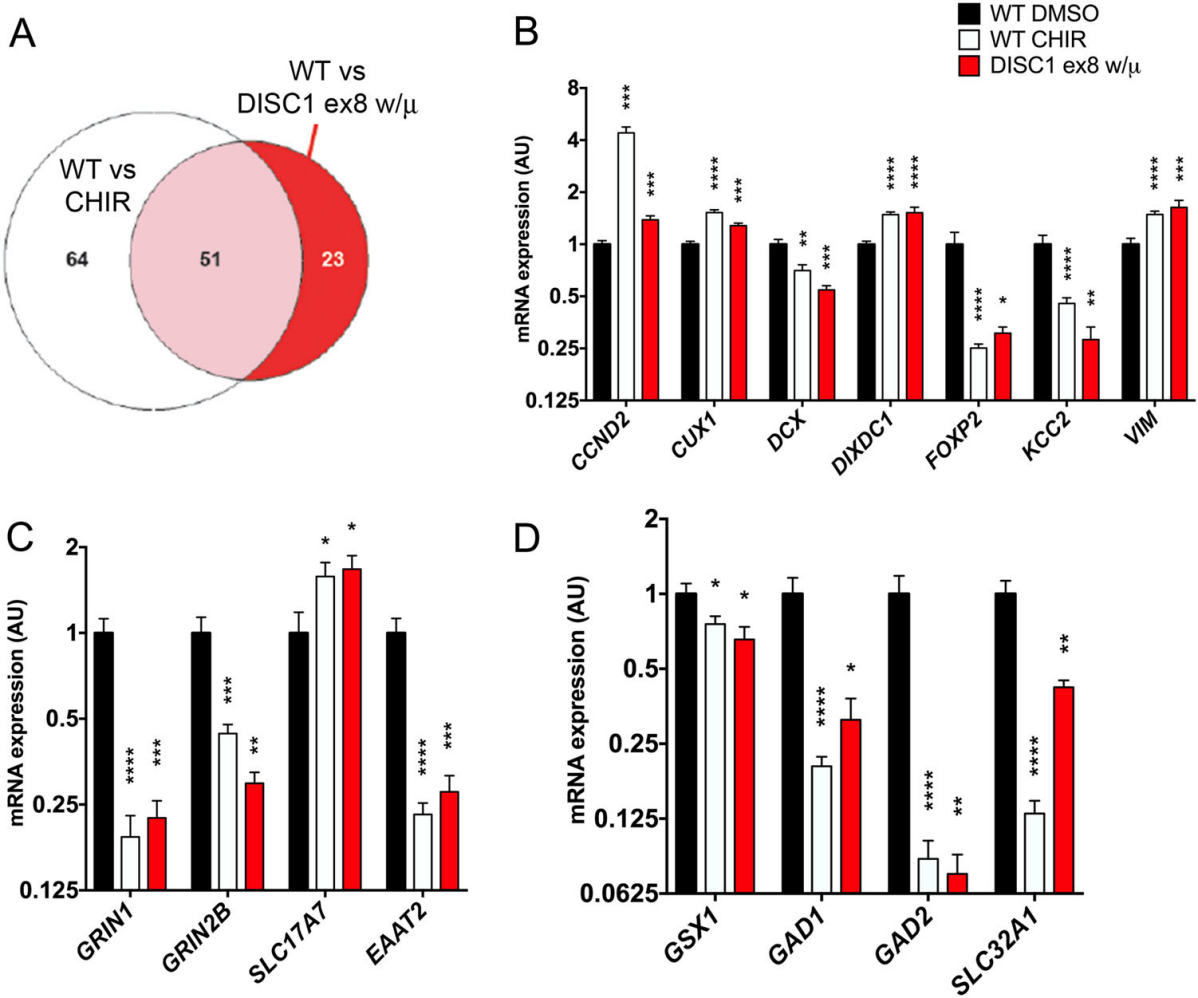
*DISC1* disruption alters expression of genes implicated in neurodevelopment and migration and is phenocopied by WNT agonism. Wild-type and *DISC1* ex8 wt/μ iPSCs were differentiated to NPCs and treated with vehicle (DMSO) or WNT agonist CHIR99021 (CHIR) during days 7–17, followed by withdrawal of small molecules and subsequent monolayer culture. Day 40 neuronal RNA was harvested and used for Nanostring. **a** Venn diagram showing genes with significantly altered expression with shared direction of fold-change compared to wild-type. Number of genes per category is shown within the diagram. Statistics: Student’s *t*-test with multiple comparisons correction using 2-stage linear step-up procedure of Benjamini, Krieger, and Yekutieli, with *Q* = 5%. **b**–**d** Nanostring data for select genes are shown, including genes associated with cell fate (**b**), genes associated with glutamatergic neurotransmission (**c**), and genes associated with interneuron development (**d**). Statistics: Holm–Sidak; WT *n* = 15, WT CHIR *n* = 18, *DISC1* ex8 wt/μ *n* = 7. *p < 0.05, **p < 0.01, ***p < 0.001, ****p < 0.0001. See also Supplementary Table 2

Given the variability of organoid cultures, we complemented this assay with a study of neuronal cells differentiated via a similar protocol but with plating of neuroepithelial cells in a monolayer^9^. Previous reports have shown that non-neural cells can arise using the protocol on which our organoid protocol is based, which could be contributing to our observed variability^6^. An important difference between our organoid and monolayer protocol is the addition of a purification step to separate the neural from non-neural cells via adherence to Matrigel followed by rosette selection. Indeed, this reduced the heterogeneity in the cell types present between individual cultures, therefore reducing variability at the gene expression level (Fig. 5e, Supplementary Table 2). Interestingly, of the genes highlighted in Fig. 5b, *BRN2*, *EEAT2*, and *NRG1* expression all were significantly reduced with DISC1 mutation in this monolayer system (Fig. 4e and ref. ^7^). A summary of organoid qPCR data (Fig. 4c, d and data not shown), as well as monolayer day 40 Nanostring data (Fig. 4e) is shown in Fig. 4f.

As WNT agonism phenocopied the organoid morphology of *DISC1* disruption, we sought to investigate the shared effects of *DISC1* mutation and WNT agonism on gene expression. Neuroepithelial aggregates were cultured with WNT agonist CHIR99021 during days 7–17 of differentiation, followed by withdrawal of the WNT agonist and neuronal culture in traditional differentiation media until day 40^7^. Changes observed at day 40 therefore represent long-lasting changes in cell state that persist more than 20 days after cessation of WNT agonism. Day 40 RNA was harvested and used for the Nanostring assay. A selection of gene expression changes observed under these conditions was published previously^7^, but here we sought to more globally evaluate changes in gene expression that were shared between *DISC1* disruption and WNT agonist treatment. Expression of a subset of general markers of neuronal and astrocyte fates were unchanged with *DISC1* mutation or CHIR99021 treatment (Supplementary Table 2, ref. ^7^). *DISC1* exon 8 mutation altered expression of 74 genes, whereas early CHIR99021 treatment of wild-type cells changed expression of 115 genes (Fig. 5a). Strikingly, 51 shared genes were significantly altered with the same directional fold-change from wild-type in each condition. This represents 69% of all genes altered with *DISC1* disruption. The marked overlap of gene expression changes with WNT agonism and *DISC1* mutation further support a model in which *DISC1* disruption leads to downstream changes in neural cells via augmented WNT signaling.

Gene expression changes shared between *DISC1* ex8 wt/μ and CHIR99021-treated wild-type cells included many genes important for neurodevelopment and cell fate. A handful of these were published previously (including *BRN2 (POU3F2), DAB2*, *FOXG1*, *GSX2*, *HES1*, *IRX3*, *SIX3*, *TBR2*, and *WNT3A*)^7^. Of those genes that showed a suggestion of alteration in day 19 organoids, *BRN2* and *EAAT2* were also decreased in neurons with *DISC1* mutation and in wild-type neurons exposed to CHIR99021 (*EAAT2* shown in Fig. 5c; *BRN2* in ref. ^7^, normalized data in Supplementary Tables 1 and 2). *DISC1* mutation and CHIR99021 treatment also led to decreased expression of lower-layer cortical neuronal marker *FOXP2* and increased expression of upper-layer marker *CUX1* (Fig. 5b). Decreased expression of the immature neuronal marker *DCX* and increased expression of pro-proliferative protein *CCND2* and neural progenitor marker Vimentin (*VIM*) further pointed to alteration in neural progenitor fates with *DISC1* mutation at this later developmental stage (Fig. 5b). We also observed increased expression of *DIXDC1*, a regulator of neurogenesis (Fig. 5b). Expression of glutamatergic cell markers showed decreased *GRIN1* and *GRIN2B* expression, but increased *VGLUT1* (*SLC17A7*) expression (Fig. 5c). Interestingly, expression of ventral progenitor markers *GSX2*^7^ and *GSX1*, and GABAergic cell markers *GAD1*, *GAD2*, and *VGAT* (*SLC32A1*) were decreased, suggesting perturbed interneuron development (Fig. 5d). Recapitulation of *DISC1* ex8 wt/μ expression changes with WNT agonism in wild-type cells was consistent with increased WNT signaling in *DISC1*-mutant lines causing cell fate and morphologic phenotypes. Overall, these data show that *DISC1* disruption alters expression of select genes related to neural development and migration in cerebral organoids and monolayer neurons, many of which are induced by WNT agonism in wild-type cells.

## Discussion

The continual evolution of stem cell differentiation methods provides opportunities for investigating human neuropsychiatric disorders in cellular models that recapitulate different aspects of human neurodevelopment. The development of 3D culture methods has allowed the study of disease-linked genetic insults on cellular organization in a model of human cortex. Here, we used this method to investigate effects of a disease-relevant *DISC1* mutation in early cerebral organoids. Analyses of organoid morphology showed that *DISC1* disruption resulted in disorganization of organoid structure, with an increased number of small and disorganized rosettes in place of the larger rosette and ventricle-like structures in wild-type organoids. This finding highlights the promise of using cerebral organoids for disease modeling, allowing the discovery of morphological phenotypes that cannot be as easily studied with monolayer cultures. This morphologic change was induced in wild-type cells with a WNT agonist, and was partially rescued with a WNT antagonist, suggesting that abnormal WNT signaling in *DISC1-*disrupted cells contribute to the morphologic phenotype. This disrupted structural organization could represent a developmental defect in neural progenitor proliferation and/or migration. Given that patients with heterozygous *DISC1* disruption do not have gross cortical structural defects^17,18^, this phenotype may be amplified in the organoid system due to the inherent qualities of the system, such as a lack of compensatory mechanisms present in vivo that are lacking in vitro (for example, mechanisms mediated by surrounding support cells such as microglia and cells of the vasculature). Furthermore, protective alleles may exist that prevent manifestation of the neurodevelopmental defects seen in this cell culture system.

The linkage between *DISC1*, WNT signaling, and NPC proliferation described here in this 3D human neurodevelopmental system is consistent with a number of findings in human subjects and mouse models of *DISC1* disruption. In both human and mouse cells, *DISC1* variants associated with mental illness reduce NPC proliferation and affect responses to WNT^12^. In both animal models and human imaging studies, findings are consistent with our data that *DISC1* disruption perturbs neurogenesis. Several studies have shown a reduction in cortical volume, enlarged ventricles, and/or thinning of the cortex in animal models with DISC1 disruption^19–23^. In addition, multiple mouse studies have shown specific reductions in GABAergic neurons in the cortex with DISC1 mutation^19,20,24,25^. Furthermore, imaging studies in humans have reported linkages between *DISC1* variants and cortical thickness in children and adolescents^26^ and in gray matter density and/or cortical thickness in adults^27–33^. In a recent imaging study of subjects in the Scottish pedigree harboring the translocation that disrupts *DISC1*, cortical thickness was significantly reduced in translocation carriers, a phenotype which was shared with SCZ subjects^17^.

We found that expression of *BRN2* was significantly reduced with a *DISC1* disruption in both cellular systems (Fig. 4). In organoids, this reduction was rescued by a WNT antagonist and phenocopied by a WNT agonist. BRN2 is a transcription factor that upregulates proneural genes, and has been shown to be required for generation and migration of upper-layer neurons in the rodent neocortex^16,34–36^. *BRN2* directly modulates expression of *TBR2*^35^, consistent with our previous finding that a *DISC1* mutation reduces production of TBR2+ NPCs in monolayer culture^7^. A genetic interaction between *BRN2* and major mental illness has been previously described: SNPs in the *BRN2* locus were significantly associated with an interaction between an imaging quantitative trait and schizophrenia diagnosis^37^. In addition, multiple GWAS studies have shown an association between SNPs near *BRN2* and bipolar disease^38–40^.

A recent study of human iPSC-derived NPCs from autistic individuals with macrencephaly showed reductions in WNT signaling, increases in proliferation and a reduction in *BRN2* levels^41^. Dysregulation of a *“β-catenin/BRN2/TBR2* transcriptional cascade” in mice was previously shown to lead to autism-related behavioral changes, as well as to a transient increase in embryonic brain size^42^. Our results also suggest disruption of this axis with *DISC1* mutation, but with an elevation of baseline canonical WNT signaling and reduction of NPC proliferation^7^. However, in both the autism model and our *DISC1*-mutant model, BRN2 levels are decreased, suggesting perhaps additional age-dependent and context-dependent outcomes of disruption of this axis. Together, these studies add further evidence to the hypothesis that subsets of autism and schizophrenia are diametrically opposed conditions with regards to their neurodevelopmental basis (ref. ^43^ and others).

The shared gene expression changes with *DISC1* interruption and WNT agonism suggest those genes that may be the best candidates for mediating the shared morphologic changes seen in cerebral organoids with *DISC1* mutation and with CHIR99021 treatment. Interestingly, many differentially expressed shared genes have known roles in cortical cell migration in development. Decreased expression of *DCX* (a marker of newly-generated neurons and regulator of neuronal migration^44–46^) and increased expression of Vimentin (a marker of neural precursor cells^47^) may indicate a dysregulation of neural progenitor fate and migration and could contribute to the altered morphology seen in organoids with *DISC1* disruption or CHIR99021 treatment. Expression of neuronal layer markers *FOXP2* and *CUX1* were dysregulated by *DISC1* disruption and WNT agonism. *FOXP2* expression is modulated by *POU3F2*, and it is a regulator of radial neuronal migration in the developing cortex^48,49^ and a marker of deep-layer cortical neurons in the adult brain^50,51^. Decreased expression of deep-layer cortical markers *FOXP2*^52^ and increased expression of upper-layer marker *CUX1*^53^ suggest a perturbation of neurogenesis, increasing the relative generation of upper-layer versus lower-layer neurons.

Certain markers of interneurons were differentially expressed with *DISC1* mutation and WNT agonism, and others were altered in *DISC1*-mutant organoids. The calcium-binding proteins *CALB1* and *CALB2*—altered in organoids—are both implicated in neuronal development of excitatory as well as inhibitory neurons, and are later expressed in subsets of interneurons^54–57^. Decreased expression of *GSX1* (a marker of ventral telencephalic development^58,59^) and inhibitory neuron markers *GAD1*, *GAD2*, and *VGAT* suggest disrupted interneuron development. This is of particular interest given the linkage of interneuron dysfunction to major mental illnesses^60^ and prior observed roles of DISC1 in interneurons^19,61^. NRG1, another gene altered with DISC1 mutation, is known to be a regulator of cortical interneuron migration via interaction with ERBB4 expressed in radial glia^62–64^. In addition, *NRG1* is a genetic susceptibility factor in schizophrenia^65,66^.

Expression of *DIXDC1*, a homolog of the WNT pathway genes *Axin* and *Disheveled*, was increased in neurons with *DISC1* disruption and WNT agonism. This protein has been found to regulate neurogenesis and neuronal migration via interaction with DISC1 by WNT-dependent and WNT-independent mechanisms, respectively^11^. Increased *DIXDC1* expression may represent a compensatory mechanism to regulate neurogenesis and/or migration resulting from loss of DISC1.

Another gene changed with DISC1 disruption is *EAAT2*, a major glutamate transporter in the human brain, that is expressed in neurons in the developing human cortex and in astrocytes postnatally^67,68^. Interestingly, EAAT2 polymorphisms and splice variants have been associated with schizophrenia^69–72^, supporting a role for altered glutamate trafficking. These findings may suggest a common mechanism of disease pathophysiology involving glutamate transmission, but require further study.

Together, altered expression of these genes with *DISC1 *disruption suggests aberrant human cortical neurogenesis, migration, and interneuron development. These are especially attractive targets for future investigation in a model of human neurodevelopment given prior studies showing roles for DISC1 in cortical neuronal migration and interneuron development in rodents^19,61,73–77^. This study builds on prior work from our group showing that *DISC1*-mutant neural progenitors displayed increased basal WNT signaling and alterations in cell fate and gene expression that were rescued by WNT antagonism^7^. In addition to showing these results in a novel culture system, we show the relevance of this altered signaling to a novel morphologic phenotype in *DISC1*-mutant cerebral organoids. These findings add to a growing literature implicating WNT signaling in the pathogenesis of psychiatric disease^12,78–82^.

## Electronic supplementary material

Supplemental Legends

Supplemental Figure 1

Supplemental Figure 2

Supplemental Tables 1 and 2
