# Supplementary material for: Shared effects of DISC1 disruption and elevated WNT signaling in human cerebral organoids
Source: Transl Psychiatry. 2018 Apr 12;8:77. doi: 10.1038/s41398-018-0122-x (PMC5895714; doi:10.1038/s41398-018-0122-x)
Supplement: Supplementary file 1 — Supplemental Legends [file 41398_2018_122_MOESM1_ESM.docx]

**Supplemental Figure Legends**

**Supplemental Figure 1. Quantification of morphological changes with *DISC1*-disruption and WNT agonism/antagonism.** Wild-type, *DISC1*-mutant, wild-type + WNT agonism (CHIR), and *DISC1-*mutant + WNT antagonism (XAV) organoids were harvested at d19 and morphological characteristics were quantified, blinded using ImageJ. (A) Number of ventricles normalized to total organoid area, (B) average ventricle area normalized to total organoid area, (C) average ventricle length normalized to total organoid area, and (D) total organoid area. Data were derived from 4 independent differentiations. Statistics: One-way ANOVA, *p<0.05, **p< 0.01, ***p<0.001, ****p>0.0001.

**Supplemental Figure 2. Additional RNA-based characterization of d19 organoids.**

Wild-type and *DISC1* ex8 wt/mut organoids were harvested at day 19 and RNA was used for qPCR analyses of listed genes. Data were derived from 3 independent differentiations, n=7-10 per condition. No significance was observed for any genes listed using a Student’s t-test.

**Tables**

**Supplementary Table 1.** Nanostring data set used to generate Figure 4A-B.

**Supplementary Table 2.** Nanostring data set used to generate Figure 4E, Figure 5.
