# Supplementary figures and images for: Shared effects of DISC1 disruption and elevated WNT signaling in human cerebral organoids
Source: Transl Psychiatry. 2018 Apr 12;8:77. doi: 10.1038/s41398-018-0122-x (PMC5895714; doi:10.1038/s41398-018-0122-x)

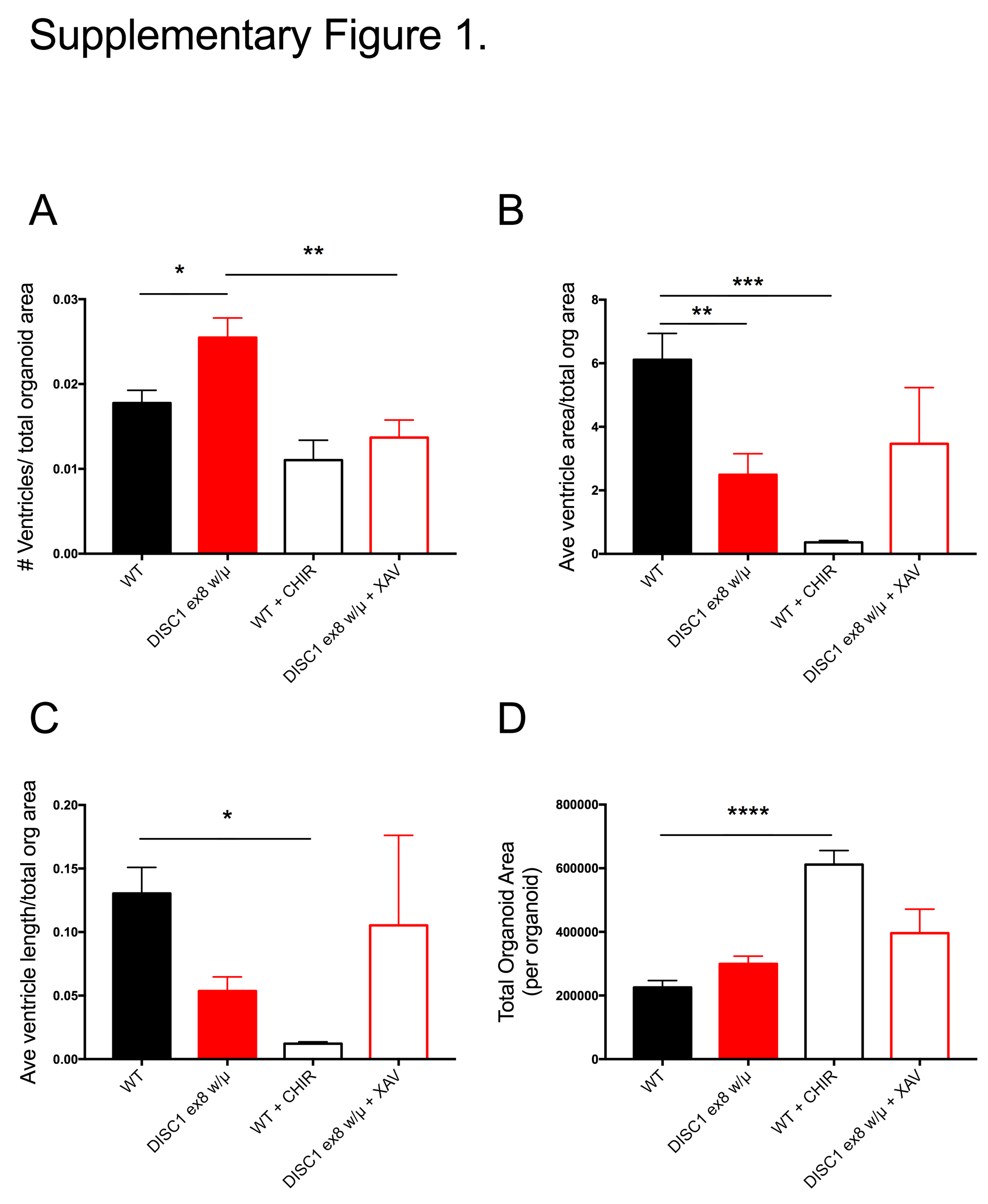

Supplement: Supplementary file 2 — Supplemental Figure 1 [file 41398_2018_122_MOESM2_ESM.tif]

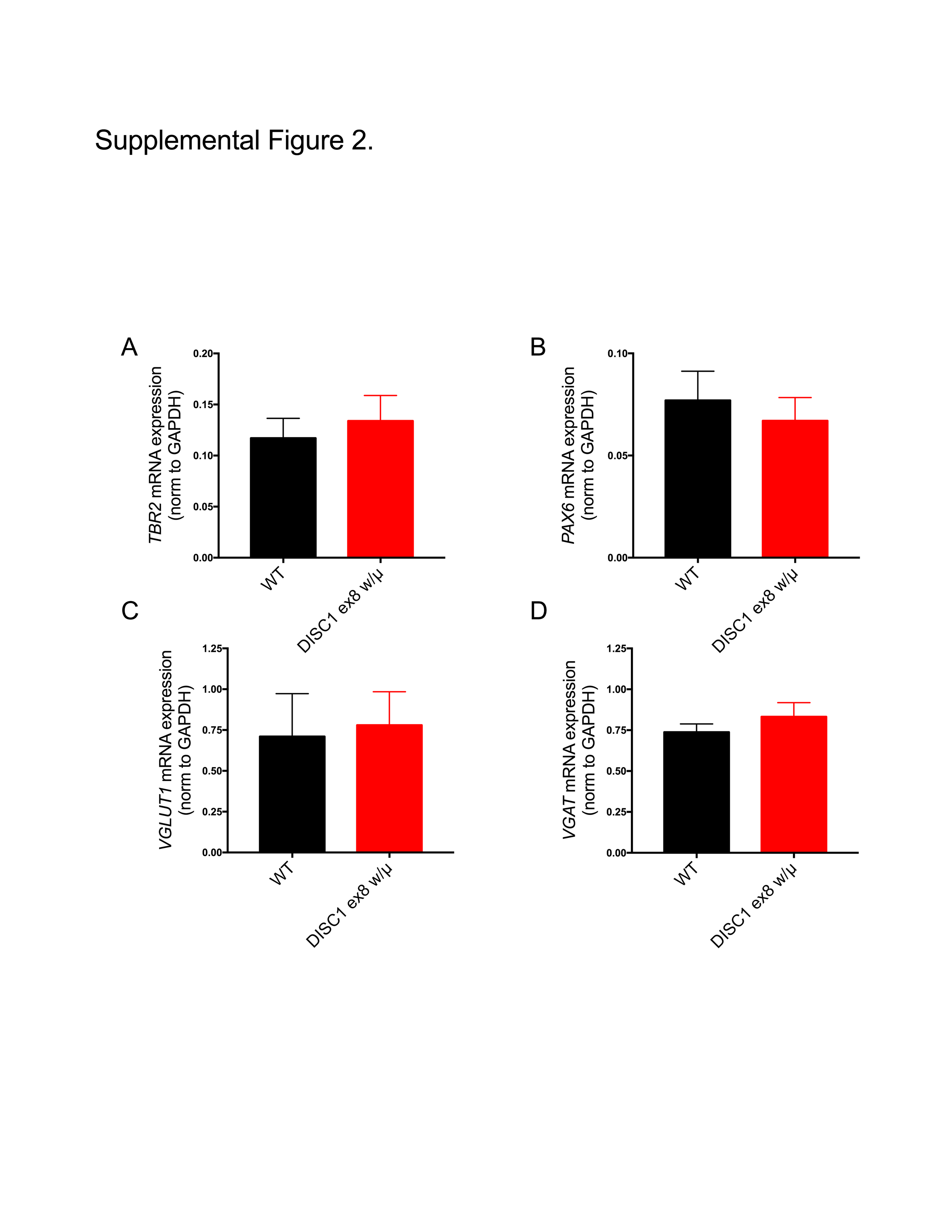

Supplement: Supplementary file 3 — Supplemental Figure 2 [file 41398_2018_122_MOESM3_ESM.tif]
