# Supplementary material for: Shared effects of DISC1 disruption and elevated WNT signaling in human cerebral organoids
Source: Transl Psychiatry. 2018 Apr 12;8:77. doi: 10.1038/s41398-018-0122-x (PMC5895714; doi:10.1038/s41398-018-0122-x)
Supplement: Supplementary file 4 — Supplemental Tables 1 and 2 [file 41398_2018_122_MOESM4_ESM.pdf]

Gene Name	Description	Wild-type organoids							DISC1 exon 8 wt/mut organoids							
OLFM1	A Domain	210	29	51	218	63	59	70	150	148	136	23	84	685	51	41
ABCA7	ABCA7	34	162	148	21	123	66	147	95	70	82	123	14	46	142	139
ADAM10	ADAM10	54	83	115	108	171	69	102	93	79	92	140	45	28	75	86
ADAM19	ADAM19	95	134	61	17	106	111	176	73	50	103	95	70	186	55	69
ADAM9	ADAM9	42	96	116	127	157	77	86	102	70	85	127	35	41	46	51
AFP	AFP	39	99	166	58	97	110	130	267	633	413	647	17	11	10	17
APLP1	APLP1	38	152	139	59	68	115	130	115	105	63	73	47	25	102	122
APLP2	APLP2	50	103	124	88	146	82	107	110	73	145	177	55	60	78	83
APOE	APOE	44	98	87	72	159	103	136	121	117	168	221	75	55	76	93
LRP8	APOER2	29	136	110	109	114	86	115	103	104	222	282	22	72	176	239
APP	APP-695	43	124	146	70	104	97	117	88	64	123	149	42	42	86	101
APP	APP-770	26	130	148	65	104	99	128	74	48	151	185	35	33	90	94
APP	APP-KPI	28	110	132	73	112	108	138	87	56	147	165	41	25	93	114
ATF3	ATF3	74	70	66	169	168	59	94	200	222	74	157	108	49	95	98
AXIN2	AXIN2	43	168	132	80	99	77	100	79	68	76	98	22	32	50	67
OLFM1	Pancortin B/M Don	77	210	109	50	68	55	132	174	231	241	128	361	585	257	395
BACE1	BACE1	69	119	117	78	130	85	103	103	74	112	121	53	48	66	72
BACE2	BACE2	22	172	176	45	108	74	103	145	128	15	99	48	3	52	64
BIN1	BIN1	32	152	126	99	107	75	109	104	105	68	134	45	1	57	85
POU3F2	BRN2	26	99	70	128	127	123	126	37	20	18	42	56	74	43	102
CCND2	CCND2	39	116	148	93	110	85	110	83	60	99	120	35	37	50	59
BCL11B	CTIP2	53	113	153	110	82	111	78	172	120	73	78	50	72	8	51
CUX2	CUX2	63	111	119	39	64	142	163	111	47	22	103	26	85	153	166
CALB1	Calbindin	70	74	108	46	170	110	121	138	239	447	843	2330	712	1329	1372
CALB2	Calretinin	87	65	52	118	116	116	146	79	66	31	52	11	119	22	7
CUX1	Cux1	66	118	130	69	106	92	119	91	94	98	139	57	30	73	84
CCND1	CyclinD1	63	109	94	86	106	118	125	116	111	111	124	125	54	111	157
DAB1	DAB1	31	78	122	155	81	90	143	99	144	11	120	13	4	107	107
DAB2	DAB2	77	108	110	102	122	68	112	107	93	91	105	59	8	32	43
DCX	DCX	58	92	116	127	90	110	107	197	165	25	20	52	78	115	123
DISC1	DISC1 L+Lv	75	157	146	27	107	87	101	42	26	26	31	31	101	41	14
DISC1	DISC1 Lv	101	148	141	135	9	102	64	91	10	33	60	12	137	96	39
DIXDC1	DIXDC1	74	117	130	103	109	53	114	112	91	65	124	88	31	74	109
SLC1A3	EAAT1	12	109	107	130	181	62	99	124	84	136	141	38	29	70	52
SLC1A2	EAAT2	55	60	67	154	97	168	98	108	25	20	5	23	75	69	43
SLC1A1	EAAT3	79	78	97	142	176	50	78	125	153	109	125	57	3	59	60
EPHA1	EPHA1	50	99	138	12	53	143	205	52	64	273	303	71	44	105	132
ERBB4	ERBB4	92	63	112	163	141	61	68	71	57	95	132	13	14	24	16
APBB1	FE65	32	165	131	73	107	79	114	123	120	103	91	63	36	86	107
FOXP2	FOXP2	148	106	82	131	91	94	48	222	200	15	44	91	57	89	78
FEZF2	Fezf2	108	38	44	39	75	227	169	135	267	681	721	146	160	553	544
FOXG1	FoxG1	131	16	62	177	64	194	56	17	13	46	103	286	16	1533	1400
GAD1	GAD1	52	120	76	19	152	102	179	52	113	182	248	178	71	159	175
GLRB	GLRB	100	123	126	110	106	75	61	419	497	66	68	272	87	221	212
GRIA2	GRIA2	107	113	134	41	122	75	109	247	205	15	69	74	5	281	176
GRIK1	GRIK1	109	125	99	29	214	77	47	52	16	36	56	59	149	39	21
GRIN2A	GRIN2A	51	149	203	41	107	58	91	44	31	176	87	19	27	31	18
GSK3B	GSK3b	19	131	134	104	127	80	105	136	140	99	133	76	22	143	151
HES1	HES1	85	124	114	91	105	81	100	124	141	60	93	86	28	72	93
HES5	HES5	24	334	101	40	76	79	47	64	104	99	101	10	3	121	107
B3GAT1	HNK1	38	176	118	18	113	123	115	299	336	24	94	234	93	270	306
HOXA1	HOXA1	132	225	168	48	23	20	84	154	153	46	199	71	179	11	51
HOXA2	HOXA2	60	160	115	67	68	100	130	126	183	26	23	117	270	25	68
HOXB13	HOXB1	80	108	73	50	124	156	109	177	36	196	129	274	109	76	119
HSPA1A	HSPA1A	18	101	91	43	124	125	199	86	74	180	136	176	122	232	262
HOXB4	HoxB4	47	96	110	94	187	63	104	97	65	116	135	47	26	16	20
IRX3	Irx3	35	179	190	59	87	71	79	65	56	135	114	5	13	33	39
ISL1	Isl1	84	76	83	174	104	97	83	275	286	30	41	98	61	86	120
PAFAH1B1	LIS1	48	77	100	161	125	83	106	146	111	105	97	64	55	101	120
LMX1A	LMX1A	26	112	112	121	140	96	93	62	97	101	130	68	111	69	66
OLFM1	M domain	96	122	100	126	91	87	79	337	360	14	44	146	14	127	127
MAP2	MAP2	27	65	87	162	149	106	103	134	185	32	45	98	27	97	115
NDEL1	NDEL1	68	96	108	113	125	93	98	97	106	118	164	79	10	92	92
NFKB1	NFKB1	9	144	141	85	105	71	145	156	144	42	129	94	23	121	148
SLC9A6	NHE6	41	140	108	85	142	76	109	75	99	39	122	74	154	118	129
SLC9A9	NHE9	78	126	137	141	78	83	58	155	171	16	56	89	69	48	42
SLC12A2	NKCC1	34	140	110	94	91	97	133	136	87	94	121	74	46	99	118
NPY	NPY	97	182	162	136	65	22	36	40	40	74	126	8	11	14	15
NRG1	NRG1	16	109	81	142	96	118	137	165	184	90	99	181	160	224	268
NES	Nestin	20	163	170	55	78	82	131	100	88	83	115	61	20	116	152
NEUROD1	NeuroD1	109	44	60	224	134	65	64	440	457	18	11	138	82	80	102
NEUROG1	Neurogenin1	84	108	53	49	120	137	149	515	386	8	28	158	32	223	301
POU5F1	Oct4	2	39	42	1	65	220	331	18	53	378	659	362	98	237	286
PDE4B	PDE4B	249	93	88	71	91	21	88	109	87	9	41	52	40	28	49
PICALM	PICALM	75	102	101	127	150	69	76	131	118	86	115	77	94	85	94
DLG4	PSD-95	41	145	124	79	109	94	107	115	107	104	144	68	21	126	158
PSEN1	PSEN1	52	128	134	76	106	93	111	103	100	88	120	78	48	125	133
PSEN2	PSEN2	45	138	133	77	154	46	108	200	247	78	111	98	189	114	131
RELN	Reelin	25	150	195	55	109	70	96	45	21	146	124	12	24	14	11
S100B	S100b	241	66	66	209	71	20	27	199	260	1	12	242	0	14	14
SORL1	SORLA	8	148	139	17	95	126	167	32	28	226	205	64	34	134	162
SYP	SYP	48	126	120	17	38	226	124	212	152	49	16	20	65	287	308
SATB1	Satb1	60	82	101	118	126	98	116	126	99	110	123	68	40	93	126
SIX3	Six3	113	73	95	30	55	168	166	93	177	37	161	61	322	1232	1602
SOX1	Sox1	35	96	69	13	105	166	217	59	65	122	294	47	99	130	210
SOX2	Sox2	28	101	67	58	100	158	190	106	129	172	250	206	97	438	591
SYN1	Synapsin	496	71	22	29	8	48	25	39	54	65	33	83	390	12	4
ADAM17	TACE/ADAM17	43	99	106	124	143	86	100	117	107	91	136	91	55	117	145
MAPT	TAU 3R	67	155	114	64	74	115	110	165	141	46	60	51	16	68	101
MAPT	TAU ALL	35	144	138	68	70	130	116	143	143	43	46	47	76	69	123
TSNAX	TSNAX ex3/4	116	80	74	178	167	41	44	133	159	69	84	115	107	61	57
TWIST1	TWIST1	117	111	83	173	94	54	67	47	44	94	96	46	75	8	14
TBR1	Tbr1	214	110	87	56	23	118	92	84	140	75	128	88	291	55	124
EOMES	Tbr2	64	69	50	17	65	184	252	6	35	133	205	166	95	61	58
TUBB3	Tuj1	79	97	86	116	83	115	124	352	344	14	30	138	40	95	116
SLC17A7	VGLUT1	154	172	140	40	75	37	82	233	23	54	255	63	229	330	298
SLC17A6	VGLUT2	33	93	117	169	117	90	81	195	162	53	21	40	27	84	119
VIM	Vimentin	63	157	136	83	88	78	95	112	121	76	80	80	46	71	92
WNT3A	WNT3A	109	193	65	39	110	93	92	24	16	38	85	45	148	49	35
WNT7A	WNT7A	99	105	62	47	88	127	171	70	172	376	166	74	268	64	116
OLFM1	Y domain	135	89	71	141	122	82	59	404	472	5	38	223	45	83	91
OLFM1	Z Domain	119	113	100	78	63	114	112	221	198	31	42	129	112	133	143
ZIC2	ZIC2	2	125	95	30	119	144	186	45	55	141	148	65	42	200	275
ZIC3	ZIC3	20	91	62	56	132	170	168	32	39	226	287	209	112	159	230
GPHN	gephyrin	25	102	128	132	120	85	109	119	129	102	131	101	13	89	117
GLUL	glutamine syntheta	71	103	120	95	134	77	100	116	114	92	111	64	41	69	77
NANOG	nanog	40	51	53	14	44	200	299	3	26	625	846	411	54	332	335
TP53	p53	47	166	114	73	78	91	130	97	93	105	108	96	55	119	132
TP73	p73	147	115	87	144	25	58	124	19	15	254	256	260	18	198	180

Gene Name	Description	Wild-type DMSO															Wild-type CHIR99021																		DISC1 exon 8 wt/mut DMSO						
OLFM1	A Domain	0.6	0.6	0.8	0.8	0.9	0.6	1.2	1.3	0.6	0.8	1.7	1.9	1.3	0.9	1.0	0.7	0.7	0.7	0.6	0.7	0.5	0.8	1.0	0.6	0.6	1.0	0.9	0.9	0.5	0.7	0.6	0.8	0.4	1.1	1.2	1.1	1.3	1.6	2.3	0.2
ABCA7	ABCA7	1.0	1.0	1.0	1.1	1.0	1.1	0.9	0.6	1.5	1.2	0.7	0.6	1.2	1.1	1.0	1.2	1.3	1.4	2.1	2.1	2.3	1.9	1.2	1.7	1.2	1.0	1.2	1.5	1.6	1.1	1.9	1.4	1.4	1.0	1.1	1.3	1.4	1.0	1.1	2.0
ADAM10	ADAM10	0.9	0.8	0.9	0.8	0.9	0.9	0.7	0.7	1.5	1.6	0.8	0.8	1.1	1.2	1.4	2.6	2.5	2.3	1.5	1.5	1.5	1.8	1.5	2.1	2.1	1.5	1.7	2.4	2.8	2.2	1.9	1.8	2.7	1.2	1.2	1.1	1.2	1.3	1.4	1.9
ADAM19	ADAM19	0.9	0.9	1.0	0.9	0.9	1.0	1.1	0.9	1.5	1.4	0.8	0.8	1.4	0.8	0.8	3.0	2.7	2.8	4.4	4.5	4.7	4.5	3.9	4.7	4.1	4.1	4.3	5.7	3.7	3.4	5.5	5.2	3.7	1.9	2.2	1.9	2.1	2.1	2.4	1.3
ADAM9	ADAM9	0.9	0.8	0.9	0.7	0.8	0.7	1.2	1.1	1.1	1.1	0.8	0.9	1.1	1.4	1.4	1.5	1.4	1.5	2.8	2.5	2.6	2.8	2.0	3.8	3.5	1.9	2.2	2.8	3.2	2.8	3.8	3.1	5.0	1.3	1.4	1.3	1.1	1.1	1.1	2.3
AFP	AFP	0.4	0.4	0.2	0.5	0.1	3.1	0.1	3.2	0.0	0.0	4.1	2.8	0.1	0.0	0.0	0.3	0.0	0.0	0.2	0.2	0.2	0.2	0.1	0.1	0.4	0.6	0.2	1.0	9.3	0.6	5.9	19.9	0.0	0.0	0.1	0.0	0.1	0.0	0.0	0.0
APLP1	APLP1	1.0	1.0	1.1	0.8	0.8	0.7	0.8	0.8	1.1	1.1	1.0	1.1	1.0	1.3	1.4	0.8	0.8	0.8	0.8	0.9	0.9	1.0	0.9	0.7	0.7	0.9	0.8	0.7	1.0	0.8	0.8	0.8	0.8	0.8	0.8	0.8	0.8	0.9	0.9	1.0
APLP2	APLP2	0.9	0.8	0.9	0.8	0.9	0.8	1.1	1.0	1.3	1.3	1.0	1.0	1.1	1.1	1.2	2.2	2.2	2.2	2.9	2.7	2.6	1.7	1.4	2.2	2.1	1.9	2.0	2.3	2.4	1.9	2.1	2.1	2.2	1.3	1.3	1.2	1.5	1.5	1.5	2.1
APOE	APOE	0.4	0.4	0.3	1.2	1.0	1.0	0.5	0.4	1.9	1.9	0.9	1.0	1.9	1.3	1.0	6.0	5.1	4.8	5.2	4.5	5.2	5.3	3.5	5.0	4.2	4.2	5.1	5.3	5.5	4.1	5.4	4.9	5.1	1.2	1.3	1.1	2.1	1.7	1.9	2.7
LRP8	APOER2	0.7	0.7	0.8	0.8	0.7	0.8	0.9	0.9	1.5	1.7	0.7	0.6	1.0	1.6	1.8	0.6	0.6	0.6	0.5	0.6	0.6	0.6	0.6	0.6	0.7	0.5	0.5	0.5	0.6	0.5	0.5	0.5	0.6	1.2	1.2	1.1	0.7	0.9	1.0	1.3
APP	APP-695	0.8	0.8	0.8	0.7	0.8	0.7	0.9	0.9	1.3	1.5	1.0	1.1	1.1	1.3	1.4	1.5	1.4	1.5	2.0	2.1	2.0	2.5	2.0	3.3	3.2	2.4	2.6	3.3	2.9	2.4	2.7	2.6	3.0	1.3	1.3	1.1	1.3	1.4	1.4	1.5
APP	APP-770	0.7	0.6	0.7	0.9	0.9	0.9	0.9	0.9	1.5	1.7	1.0	1.0	1.2	1.1	1.1	4.4	4.2	4.3	5.8	5.8	5.9	8.0	5.7	12.3	12.1	7.0	8.1	11.7	11.0	9.1	9.0	8.7	11.1	1.6	1.7	1.5	2.1	2.3	2.4	2.3
APP	APP-KPI	0.7	0.7	0.7	0.8	0.8	0.8	0.9	0.8	1.4	1.6	0.9	1.0	1.3	1.3	1.3	2.1	2.0	2.1	3.2	3.2	3.4	3.7	2.7	5.0	4.8	3.4	3.8	5.2	3.9	3.5	4.2	4.1	4.6	1.4	1.5	1.3	1.6	1.8	2.1	1.9
ATF3	ATF3	0.6	0.5	0.6	0.6	0.6	0.7	0.7	0.6	1.8	2.1	0.4	0.5	2.2	1.4	1.6	1.1	1.1	1.1	1.3	1.3	1.1	0.4	0.4	0.5	0.6	0.6	0.7	0.8	0.9	0.8	0.9	0.9	0.6	1.8	1.7	1.3	1.6	1.8	1.6	3.8
AXIN2	AXIN2	1.1	1.1	1.2	0.8	0.9	0.8	1.6	1.5	0.8	0.8	1.4	1.3	0.6	0.6	0.6	2.4	2.3	2.3	5.1	5.0	4.9	2.3	1.9	2.6	2.6	3.3	3.5	2.4	2.5	2.1	4.2	3.7	2.5	1.1	1.2	1.3	1.1	1.0	1.2	1.0
OLFM1	B/M Domain	0.6	0.6	0.7	0.9	0.9	0.8	0.8	0.9	0.8	1.0	1.4	1.6	1.1	1.3	1.5	0.5	0.5	0.7	0.5	0.6	0.6	0.6	0.6	0.4	0.4	0.6	0.6	0.6	0.5	0.6	0.5	0.4	0.5	1.3	1.3	1.1	0.9	1.5	1.7	0.4
BACE1	BACE1	1.0	1.0	1.1	1.0	1.2	1.0	0.8	0.7	1.1	1.3	0.7	0.7	0.9	1.1	1.4	2.4	2.3	2.3	1.9	1.9	1.8	2.2	1.9	2.4	2.3	1.8	2.0	2.1	2.6	2.2	2.0	2.0	2.6	1.3	1.3	1.1	1.0	1.2	1.3	2.1
BACE2	BACE2	0.9	0.8	0.7	1.1	1.0	0.9	1.4	1.2	0.9	0.8	1.3	1.3	0.9	1.0	0.8	13.3	11.6	10.0	6.6	7.0	6.2	8.0	6.0	7.1	6.9	7.4	8.2	8.6	8.5	8.5	11.8	9.8	13.2	1.0	1.1	1.0	1.5	1.4	1.5	1.3
BIN1	BIN1	0.6	0.6	0.6	1.0	0.9	0.9	0.6	0.5	1.9	1.7	0.7	0.7	1.9	1.4	1.2	1.4	1.4	1.3	1.1	1.2	1.3	2.2	1.7	2.8	2.9	1.9	2.0	3.2	3.4	2.5	1.7	1.7	3.0	0.9	0.9	0.9	1.3	1.3	1.4	1.2
POU3F3	BRN1	1.0	1.1	1.1	1.1	1.2	1.0	0.8	0.9	0.9	0.7	1.3	1.3	1.2	0.7	0.7	0.0	0.0	0.0	0.0	0.0	0.0	0.0	0.1	0.0	0.0	0.0	0.0	0.0	0.0	0.0	0.0	0.0	0.0	0.8	0.9	0.8	1.0	1.2	1.3	0.4
POU3F2	BRN2	0.9	0.9	0.9	0.7	0.8	0.7	0.7	0.6	1.1	1.0	0.8	0.8	1.2	1.9	2.1	0.1	0.1	0.1	0.1	0.1	0.1	0.2	0.2	0.1	0.0	0.1	0.1	0.1	0.1	0.1	0.1	0.1	0.1	0.5	0.5	0.6	0.4	0.5	0.5	0.7
CCND2	CCND2	1.1	1.1	1.2	1.1	1.1	1.1	1.0	0.8	1.3	1.3	0.8	0.8	0.8	0.8	0.8	2.2	2.1	2.1	4.6	4.3	4.1	4.3	3.1	5.2	4.5	3.3	4.1	4.9	5.7	4.9	6.8	5.5	7.7	1.3	1.3	1.4	1.4	1.3	1.3	1.8
CHAT	CHAT	0.9	0.9	1.1	0.9	1.0	1.0	0.8	0.9	1.4	1.2	0.7	0.8	1.0	1.0	1.4	1.2	1.0	0.9	1.0	1.4	1.3	0.8	0.8	1.5	1.0	1.3	1.0	1.6	1.1	1.0	1.6	1.0	4.5	1.5	1.5	1.4	1.4	1.2	1.3	1.2
COX2	COX2	0.5	0.5	0.6	0.7	0.6	0.8	0.4	0.5	2.8	4.6	0.3	0.4	1.2	0.5	0.6	0.9	1.0	0.9	2.4	2.6	2.8	1.2	1.4	1.1	0.6	3.0	2.7	2.3	1.7	1.2	3.9	4.3	0.9	3.0	3.4	2.0	2.2	2.5	2.7	1.7
BCL11B	CTIP2	1.7	1.8	1.9	0.5	0.3	0.4	2.2	2.1	0.4	0.3	1.2	1.1	0.3	0.4	0.3	0.3	0.3	0.3	0.9	0.9	0.9	0.3	0.2	0.4	0.3	0.7	0.8	0.7	0.3	0.3	0.5	0.4	0.4	0.5	0.5	0.8	0.4	0.3	0.3	0.2
CUX2	CUX2	1.4	1.4	1.5	0.9	0.8	1.0	1.6	1.5	0.6	0.5	1.1	1.1	0.6	0.5	0.5	0.7	0.8	0.7	1.6	1.5	1.4	1.2	1.1	1.0	1.1	1.7	1.8	1.2	0.8	0.7	1.0	1.1	0.9	1.0	0.9	1.2	0.7	0.6	0.7	0.5
CALB1	Calbindin	0.6	0.6	0.6	0.6	0.5	0.5	1.5	1.8	0.4	0.4	1.9	2.1	2.1	0.6	0.7	0.6	0.6	0.7	0.7	0.8	0.8	0.5	0.4	0.8	1.0	0.9	1.1	1.4	0.6	0.8	0.9	0.9	0.5	1.1	1.0	0.9	1.6	1.8	1.9	1.5
CALB2	Calretinin	0.6	0.6	0.6	0.5	0.3	0.4	3.0	2.8	0.5	0.4	0.8	0.8	0.6	1.7	1.4	0.3	0.2	0.2	1.4	1.6	1.6	0.9	1.0	0.6	0.5	1.8	2.3	0.4	0.8	0.6	3.4	3.1	0.5	1.0	1.0	1.1	0.3	0.3	0.4	0.3
CUX1	Cux1	1.1	1.1	1.1	0.8	0.9	0.9	0.9	0.9	1.1	1.2	0.8	0.8	0.9	1.2	1.3	1.5	1.5	1.3	1.4	1.4	1.4	1.4	1.1	1.7	1.7	1.4	1.4	1.9	1.8	1.5	1.4	1.4	2.2	1.3	1.3	1.2	1.1	1.3	1.3	1.4
CCND1	CyclinD1	0.6	0.6	0.7	0.8	0.7	0.8	0.9	0.9	1.5	1.7	1.1	1.1	1.6	1.0	1.1	0.6	0.6	0.6	1.2	1.2	1.2	1.3	1.2	0.8	0.8	1.4	1.4	1.0	0.9	0.7	1.2	1.1	0.8	1.2	1.2	1.1	1.4	1.6	1.6	1.2
DAB1	DAB1	2.0	1.9	2.0	1.0	0.7	0.8	1.3	1.2	0.4	0.3	1.1	1.1	0.4	0.4	0.4	0.2	0.2	0.2	0.4	0.3	0.4	1.4	0.9	2.7	2.2	0.7	0.8	1.5	0.3	0.3	0.6	0.5	1.2	0.4	0.4	0.4	0.3	0.3	0.4	0.2
DAB2	DAB2	0.5	0.5	0.6	0.5	0.5	0.5	1.3	1.2	2.7	2.2	1.0	1.0	1.2	0.8	0.6	4.2	4.2	4.2	9.5	9.1	9.6	11.4	8.9	7.8	6.9	8.9	9.9	6.7	7.0	5.4	12.7	11.2	8.0	2.4	2.8	2.4	2.8	2.7	3.3	1.6
DCX	DCX	1.3	1.3	1.5	0.9	0.8	0.9	1.0	0.9	0.7	0.6	1.1	1.1	0.6	1.1	1.2	0.8	1.0	0.9	0.8	1.0	0.9	1.1	1.0	0.4	0.4	0.7	0.7	0.5	0.5	0.5	0.5	0.5	0.4	0.6	0.6	0.7	0.5	0.5	0.5	0.4
DISC1	DISC1 L+Lv	1.1	1.1	1.3	1.2	1.1	0.8	1.5	1.4	0.7	0.5	1.3	0.9	0.7	0.7	0.5	5.8	5.6	4.4	5.1	5.4	4.6	5.3	4.2	5.9	5.9	5.4	5.4	6.4	5.1	4.8	4.9	4.9	6.5	0.5	0.6	0.5	0.7	0.7	0.6	0.5
DISC1	DISC1 Lv	1.4	1.4	1.3	1.0	1.1	0.8	1.4	1.2	1.0	0.9	0.9	0.9	0.6	0.7	0.6	4.1	4.1	4.4	3.9	3.3	3.1	3.2	2.5	4.0	3.6	3.0	2.8	3.7	3.6	2.9	3.1	3.2	4.6	0.6	0.7	0.7	0.6	0.7	0.4	0.9
DISC1	DISC1 d3	0.7	0.7	0.8	0.7	0.7	0.6	0.9	0.9	2.0	2.1	0.4	0.4	1.1	1.4	1.4	3.2	2.7	3.3	2.0	2.2	2.2	1.7	1.7	3.0	2.8	1.2	1.5	1.4	2.8	2.4	2.2	2.3	3.2	1.4	1.4	1.4	1.2	1.6	0.9	1.9
DISC1	DISC1 esv1	1.1	1.2	0.5	0.7	1.2	1.0	1.2	0.9	1.5	1.6	0.9	0.7	1.1	0.4	1.1	3.2	3.0	1.9	2.1	2.7	3.1	3.7	2.8	4.8	4.0	2.3	2.2	2.6	2.1	1.7	2.1	2.1	5.0	1.5	1.3	1.9	0.7	0.9	1.4	1.9
DISC1	DISC1 ex1/2	1.1	0.9	0.9	1.1	1.3	1.0	1.0	1.2	1.4	0.9	1.0	0.6	1.0	0.6	1.1	2.9	1.9	3.0	2.7	2.4	3.1	3.9	2.7	4.2	4.1	3.3	3.2	5.3	3.9	2.4	3.6	3.1	4.4	1.3	1.1	1.0	1.5	1.5	1.7	1.5
DISC1	DISC1 s	0.5	0.3	1.0	0.5	1.2	1.2	1.5	1.0	2.8	1.9	0.3	0.3	0.8	0.6	0.9	5.1	4.4	4.0	3.5	5.2	5.0	4.6	4.7	7.3	5.8	1.9	1.5	3.5	2.8	2.4	3.3	2.2	9.0	1.1	1.3	1.3	1.1	0.5	0.8	2.4
DISC1	DISC1_ex6-9	0.6	0.7	0.1	0.4	0.6	0.8	0.9	0.7	3.0	3.5	0.1	0.2	0.9	1.1	1.4	3.1	2.9	2.1	1.8	1.6	1.7	1.3	1.3	2.1	1.8	0.8	1.1	0.8	1.7	2.0	1.7	1.4	2.3	1.8	1.5	1.5	0.7	1.3	0.5	2.4
DIXDC1	DIXDC1	0.9	0.9	1.0	0.9	1.2	0.9	0.9	1.0	1.0	1.4	0.9	1.0	1.1	0.7	1.1	1.6	1.5	1.6	1.3	1.4	1.3	1.3	1.2	1.6	1.7	1.4	1.4	2.0	1.9	1.7	1.2	1.3	1.4	1.4	1.4	1.1	1.3	1.8	1.8	1.9
SLC1A3	EAAT1	2.1	2.1	2.4	1.1	1.0	0.8	1.1	1.0	0.3	0.2	0.9	0.9	0.3	0.4	0.3	0.4	0.4	0.3	1.5	1.5	1.4	1.2	0.9	0.4	0.4	0.9	0.9	0.3	0.6	0.5	1.7	1.4	1.3	0.5	0.5	0.5	0.4	0.5	0.5	0.2
SLC1A2	EAAT2	1.5	1.5	1.6	0.7	0.8	0.7	1.5	1.3	0.3	0.4	1.5	1.5	0.4	0.6	0.7	0.3	0.4	0.3	0.3	0.3	0.3	0.2	0.2	0.1	0.1	0.2	0.2	0.2	0.1	0.2	0.2	0.2	0.1	0.4	0.3	0.4	0.2	0.2	0.2	0.2
SLC1A1	EAAT3	1.7	1.8	1.9	1.0	0.8	0.7	0.9	0.9	0.5	0.4	1.2	1.2	0.4	0.9	0.7	1.3	1.2	1.2	1.6	1.6	1.4	1.4	1.2	1.5	1.4	1.1	1.3	1.5	1.7	1.6	1.5	1.5	1.9	0.4	0.4	0.5	0.4	0.4	0.4	0.5
EN1	EN-1	1.3	1.0	1.3	0.8	1.1	0.8	0.7	0.7	1.3	1.3	0.7	0.8	0.9	0.9	1.2	29.2	32.8	33.3	47.9	46.6	44.5	72.6	50.0	98.8	84.6	46.7	56.8	76.0	36.7	29.8	69.8	57.0	111.6	2.5	3.0	2.2	1.3	1.2	1.1	1.0
ERBB4	ERBB4	0.8	0.8	0.9	0.4	0.4	0.6	1.5	1.3	1.5	1.0	1.0	0.8	1.4	1.3	1.3	0.4	0.4	0.4	0.8	0.8	0.7	0.2	0.2	0.4	0.4	0.4	0.4	0.4	0.2	0.2	0.3	0.3	0.5	0.6	0.7	0.8	0.6	0.6	0.6	1.0
APBB1	FE65	0.9	0.9	0.9	0.9	0.8	0.8	0.7	0.7	1.2	1.3	0.9	1.0	1.3	1.2	1.3	0.9	0.9	0.9	0.9	0.9	1.0	1.0	0.9	1.1	1.0	1.0	1.0	1.1	1.1	0.9	0.9	0.9	1.2	1.0	1.0	0.9	1.0	1.1	1.1	1.0
FOSL1	FOSL1 (FRA	0.7	0.9	1.3	1.1	1.1	1.4	0.5	0.5	0.9	1.2	0.7	0.6	1.9	1.4	1.1	1.0	0.9	0.9	0.6	0.6	0.5	0.7	0.5	0.6	0.5	0.6	0.8	1.0	0.5	0.4	0.7	0.5	0.7	1.6	1.7	1.1	1.5	1.3	1.2	2.2
FOXP2	FOXP2	2.2	2.1	2.4	1.0	1.1	1.0	0.6	0.6	0.6	0.7	0.9	0.9	0.4	0.3	0.3	0.3	0.3	0.2	0.2	0.2	0.2	0.2	0.2	0.2	0.2	0.3	0.3	0.4	0.3	0.2	0.3	0.3	0.2	0.3	0.3	0.3	0.3	0.3	0.4	0.2
FEZF2	Fezf2	1.5	1.4	1.4	0.9	1.2	1.0	1.1	1.1	0.7	0.7	1.1	1.2	0.9	0.5	0.4	0.0	0.0	0.0	0.0	0.0	0.0	0.0	0.1	0.0	0.0	0.0	0.0	0.0	0.0	0.0	0.0	0.0	0.0	1.2	1.2	1.3	1.4	1.4	1.5	0.6
FOXG1	FoxG1	1.9	1.9	2.0	2.0	1.7	1.7	0.4	0.4	0.5	0.4	0.6	0.7	0.3	0.3	0.2	0.0	0.0	0.0	0.0	0.0	0.0	0.0	0.1	0.0	0.0	0.0	0.0	0.0	0.0	0.0	0.0	0.0	0.0	0.2	0.2	0.2	0.2	0.2	0.2	0.1
GAD1	GAD1	1.0	1.1	1.2	0.7	0.5	0.5	0.7	0.5	0.9	0.7	0.9	0.7	0.8	2.5	2.4	0.2	0.2	0.2	0.4	0.4	0.4	0.2	0.2	0.2	0.2	0.1	0.1	0.1	0.1	0.1	0.2	0.2	0.2	0.3	0.3	0.3	0.2	0.2	0.2	0.7
GAD2	GAD2	1.3	1.4	1.5	0.6	0.4	0.4	0.5	0.4	0.6	0.5	1.1	1.0	0.5	2.4	2.5	0.1	0.2	0.2	0.2	0.2	0.2	0.0	0.0	0.0	0.0	0.1	0.1	0.0	0.1	0.0	0.1	0.1	0.0	0.1	0.1	0.1	0.1	0.0	0.1	0.1
GLRB	GLRB	1.4	1.4	1.4	1.2	1.1	1.1	0.9	0.8	0.8	0.6	0.8	0.7	0.7	1.0	0.9	1.5	1.6	1.6	1.4	1.3	1.5	2.3	1.8	2.5	2.1	1.8	1.9	2.3	1.8	1.4	1.5	1.4	1.4	0.6	0.7	0.7	0.6	0.6	0.6	1.2
GRIA2	GRIA2	1.7	1.8	2.0	0.8	0.8	0.8	1.3	1.2	0.7	0.7	0.7	0.7	0.4	0.7	0.7	0.3	0.3	0.3	0.4	0.4	0.4	1.4	1.3	3.1	2.9	0.7	0.6	1.2	0.5	0.5	0.6	0.5	0.8	0.6	0.6	0.6	0.2	0.3	0.3	0.5
GRIK1	GRIK1	2.1	2.0	2.2	0.6	0.5	0.6	1.3	1.2	0.3	0.3	1.2	1.1	0.6	0.5	0.5	4.8	5.3	4.6	1.1	1.1	1.0	0.5	0.5	1.8	2.0	1.0	1.0	1.7	2.7	2.4	1.1	1.2	1.6	0.3	0.3	0.4	0.5	0.5	0.6	0.3
GRIN2A	GRIN2A	0.6	0.6	0.5	0.6	0.7	0.7	1.6	1.4	0.8	1.0	1.4	1.2	1.3	1.4	1.1	3.0	2.7	2.8	3.4	3.9	3.6	1.8	1.1	8.6	8.1	2.5	3.1	12.9	11.2	9.8	4.4	4.4	6.7	0.6	0.8	0.7	0.9	0.6	0.7	0.8
GRIN2B	GRIN2B	1.6	1.6	2.1	0.6	0.9	0.6	1.3	1.3	0.3	0.5	1.3	1.2	0.4	0.6	0.8	0.4	0.5	0.4	0.4	0.4	0.4	0.7	0.7	0.4	0.4	0.6	0.6	0.4	0.2	0.3	0.3	0.2	0.6	0.3	0.3	0.4	0.2	0.2	0.3	0.4
GSK3B	GSK3b	0.9	0.9	1.0	0.8	0.8	0.8	0.6	0.6	1.6	1.5	0.6	0.6	1.2	1.6	1.5	1.4	1.4	1.3	1.1	1.1	1.1	1.1	0.9	1.2	1.2	1.0	1.0	1.2	1.1	0.9	0.9	0.9	1.1	0.9	0.9	0.9	0.8	0.9	0.8	1.5
GSX1	GSX1	1.0	1.1	0.8	0.6	0.5	0.7	1.4	1.2	0.8	0.8	1.8	1.0	0.4	1.4	1.4	0.7	0.4	1.1	0.7	0.9	0.8	0.5	0.9	0.8	0.5	0.6	0.5	0.7	1.3	0.8	0.7	0.6	1.1	0.8	0.6	0.5	0.6	0.7	0.3	1.0
GSX2	GSX2	0.6	0.5	0.5	0.7	0.5	0.7	0.4	0.4	2.1	1.7	0.5	0.3	2.1	2.3	1.7	0.0	0.0	0.0	0.0	0.0	0.0	0.0	0.1	0.0	0.0	0.0	0.0	0.0	0.0	0.0	0.0	0.0	0.0	0.4	0.5	0.5	0.3	0.3	0.3	0.3
HES1	HES1	1.4	1.4	1.4	1.1	0.9	1.0	0.8	0.8	0.9	0.9	0.9	1.0	1.0	0.7	0.7	0.9	0.9	0.9	1.0	1.0	1.0	1.2	1.1	1.3	1.3	1.2	1.3	1.3	1.3	1.1	1.3	1.4	1.1	1.2	1.2	1.1	1.4	1.6	1.6	1.1
HES5	HES5	0.7	0.6	0.6	1.0	0.8	1.0	0.4	0.4	1.7	1.4	0.6	0.5	2.2	1.7	1.4	0.0	0.0	0.0	0.0	0.0	0.0	0.0	0.1	0.0	0.0	0.0	0.0	0.0	0.0	0.0	0.0	0.0	0.0	0.7	0.7	0.8	1.3	1.3	1.4	0.4
B3GAT1	HNK1	1.0	0.9	1.0	0.9	0.9	0.8	1.4	1.3	0.8	0.8	1.3	1.3	0.9	0.9	0.9	1.6	1.5	1.2	0.7	0.8	0.7	0.9	0.8	0.5	0.5	0.8	0.8	0.7	0.6	0.5	0.7	0.6	0.5	0.8	0.9	0.7	0.6	0.7	0.9	0.4
HOXA1	HOXA1	0.1	0.2	0.1	1.8	1.6	2.0	0.7	0.6	1.2	1.3	1.1	1.3	0.6	1.0	1.3	11.3	11.3	10.0	5.5	5.7	5.8	5.0	3.8	7.0	6.8	4.6	5.2	8.3	7.8	6.0	4.4	4.3	5.0	0.9	1.1	0.9	1.9	2.2	1.9	4.5
HOXA2	HOXA2	0.6	0.7	0.7	0.3	0.4	0.4	3.1	3.0	0.4	0.2	1.8	1.7	0.3	0.8	0.8	7.0	6.7	5.9	4.7	5.2	4.9	4.3	4.0	4.7	5.1	4.5	4.7	4.4	2.9	3.1	3.6	3.7	2.9	1.3	1.5	1.6	1.1	1.4	1.5	1.7
HOXB13	HOXB13	1.1	1.7	1.2	1.4	1.2	1.1	0.7	0.7	1.4	1.2	0.4	0.8	0.7	0.7	0.8	22.2	23.3	20.8	5.9	6.0	8.2	31.2	24.0	41.5	40.6	15.7	16.0	22.7	35.8	18.7	19.9	17.9	5.7	0.6	0.3	1.1	1.3	0.1	0.1	2.1
HOXB6	HOXB6	1.0	0.8	1.0	1.2	0.8	1.3	0.7	0.8	0.9	0.8	1.1	0.5	1.3	2.0	0.6	13.7	13.9	15.0	19.4	22.9	18.7	88.8	75.3	14.0	11.5	31.6	31.5	11.6	17.5	13.9	35.3	33.3	32.5	1.5	1.0	1.1	0.7	0.9	0.4	1.7
HSPA1A	HSPA1A	0.8	0.9	0.9	1.3	1.3	1.2	0.5	0.5	1.1	1.2	1.0	1.0	1.7	0.7	0.7	1.0	1.1	1.1	1.3	1.6	1.7	1.7	1.3	1.0	1.0	1.7	1.7	1.7	1.0	0.8	1.3	1.5	1.6	0.9	1.0	0.8	2.2	2.3	2.5	1.4
HOXB4	HoxB4	0.2	0.2	0.2	0.7	0.6	0.6	1.9	1.6	0.3	0.3	1.9	2.0	0.3	2.1	2.0	2.9	2.8	2.6	1.9	2.2	1.9	2.8	2.7	4.2	4.0	2.1	2.2	3.2	3.4	2.9	2.3	2.4	4.6	0.5	0.6	0.6	0.6	0.6	0.6	1.2
IRX3	Irx3	0.5	0.5	0.5	1.0	0.9	1.0	2.0	1.6	1.5	1.4	0.8	0.8	0.7	1.0	0.7	9.1	8.7	9.4	10.6	11.2	10.6	5.2	3.4	9.1	8.6	8.9	11.7	11.6	11.4	8.4	8.7	8.4	8.5	2.0	2.1	1.8	2.5	2.6	2.9	2.7
ISL1	Isl1	1.7	1.9	1.9	0.9	0.7	0.5	0.3	0.3	0.2	0.2	1.6	1.4	0.2	1.9	1.2	13.2	14.4	14.2	11.9	13.0	12.7	16.7	14.8	6.1	7.1	13.5	12.4	9.0	10.6	8.6	9.6	10.2	7.0	0.1	0.1	0.1	0.2	0.1	0.1	0.3
SLC12A5	KCC2	1.5	1.6	2.2	0.9	1.0	1.0	1.0	0.9	0.5	0.5	1.1	1.0	0.2	0.6	1.0	0.4	0.3	0.3	0.5	0.3	0.5	0.8	0.6	0.6	0.6	0.4	0.4	0.4	0.3	0.3	0.6	0.6	0.5	0.4	0.3	0.4	0.1	0.2	0.1	0.3
PAFAH1B1	LIS1	1.6	1.6	1.7	1.0	0.8	0.9	1.2	1.1	0.6	0.5	1.1	1.1	0.6	0.6	0.6	2.3	2.4	2.3	2.1	2.2	2.1	2.3	1.9	2.1	2.0	1.9	2.0	2.0	1.6	1.6	1.9	1.8	2.1	0.6	0.6	0.7	0.7	0.6	0.7	0.7
LMX1A	LMX1A	0.7	0.7	0.7	0.5	0.5	0.5	2.3	2.1	0.8	0.7	1.3	1.4	1.3	0.7	0.6	2.0	2.3	2.5	6.6	6.1	5.8	0.8	0.7	1.3	1.2	2.2	2.3	0.9	1.1	1.1	2.1	1.9	0.8	1.6	1.7	1.8	1.6	1.4	1.5	1.3
OLFM1	M domain	0.8	0.8	0.8	0.9	0.9	0.9	1.0	1.0	0.7	0.8	1.3	1.4	0.9	1.4	1.4	0.8	0.8	0.9	0.6	0.6	0.6	0.7	0.6	0.4	0.5	0.7	0.6	0.6	0.7	0.6	0.5	0.5	0.4	1.2	1.2	1.1	0.8	1.1	1.1	0.4
MAP2	MAP2	1.8	1.8	1.9	0.9	0.8	0.9	1.0	1.0	0.6	0.4	0.9	1.0	0.6	0.7	0.6	0.3	0.3	0.3	0.7	0.6	0.6	0.2	0.2	0.2	0.2	0.3	0.3	0.1	0.2	0.2	0.4	0.3	0.2	0.4	0.4	0.5	0.3	0.3	0.3	0.3
MMP7	MMP7	0.4	0.4	0.6	1.0	0.6	0.8	0.8	0.8	0.8	1.1	0.6	0.6	2.1	2.1	2.5	0.6	0.4	0.6	0.6	0.6	0.5	0.6	0.3	1.5	7.7	0.6	0.4	0.7	0.9	1.8	1.4	1.5	0.5	0.8	0.5	0.7	0.4	0.4	0.5	1.0
MYOD1	MYOD1	1.7	1.5	0.9	1.2	1.3	1.2	0.7	1.0	1.0	1.5	0.1	0.3	1.2	0.1	1.2	1.5	2.4	1.8	2.5	1.9	1.1	8.2	4.8	8.2	29.7	1.5	1.9	5.8	13.5	53.6	4.4	1.9	4.2	1.0	1.2	1.1	1.1	0.9	0.2	1.5
NDEL1	NDEL1	0.7	0.7	0.7	0.7	0.8	0.8	0.6	0.5	2.3	2.0	0.5	0.4	1.2	1.6	1.6	1.8	1.7	1.7	1.6	1.6	1.7	2.2	1.8	2.4	2.3	1.5	1.7	2.0	2.2	1.8	2.0	1.7	2.9	1.0	1.1	1.0	0.9	0.9	0.9	2.1
NFKB1	NFKB1	1.2	1.2	1.3	0.9	1.0	0.9	1.0	1.0	0.9	1.2	0.8	0.9	0.8	0.8	1.1	3.7	3.4	3.0	2.3	2.3	2.3	3.9	3.2	4.4	4.4	2.9	3.2	3.8	3.0	2.7	3.0	3.0	3.9	1.2	1.3	1.1	1.1	1.2	1.2	1.7
SLC9A6	NHE6	1.1	1.2	1.2	0.9	0.9	0.9	0.8	0.8	1.1	1.0	0.9	0.9	0.9	1.2	1.2	0.9	0.9	0.9	1.2	1.2	1.1	1.3	1.0	1.3	1.2	1.1	1.2	1.3	1.1	1.0	1.2	1.1	1.3	0.8	0.8	0.8	0.7	0.8	0.7	1.0
SLC9A9	NHE9	1.2	1.3	1.1	1.2	1.4	1.1	0.7	0.8	0.6	0.6	1.2	1.1	1.1	1.2	0.7	9.9	10.4	10.2	5.6	5.5	6.3	11.9	9.3	15.2	14.5	8.2	8.9	17.3	13.2	13.0	7.6	7.7	16.0	0.6	0.6	0.5	1.4	1.6	2.0	0.6
SLC12A2	NKCC1	0.7	0.9	0.9	0.8	1.0	0.9	0.8	0.9	1.4	1.5	0.8	0.8	1.2	1.0	1.3	2.2	2.4	2.2	2.0	2.3	2.2	2.9	2.4	2.8	2.8	2.5	2.5	3.2	1.9	1.8	1.9	2.1	2.2	1.1	1.1	1.0	1.1	1.4	1.4	1.2
GRIN1	NMDAR	1.2	1.2	1.3	1.5	1.3	1.4	1.4	1.4	0.3	0.2	1.3	1.2	0.2	0.6	0.6	0.0	0.1	0.2	0.1	0.2	0.1	0.6	0.3	0.0	0.0	0.3	0.3	0.0	0.1	0.1	0.4	0.3	0.3	0.3	0.3	0.4	0.2	0.2	0.1	0.1
NPY	NPY	0.4	0.4	0.3	0.4	0.3	0.3	1.1	1.1	0.8	0.8	1.3	1.8	1.2	2.6	2.0	0.7	0.5	0.8	0.8	0.9	0.8	1.2	0.8	18.5	11.2	1.2	1.1	10.1	1.5	1.8	0.9	1.1	15.8	1.7	1.8	1.4	1.0	1.2	1.2	2.8
NRG1	NRG1	1.1	1.1	1.1	1.0	0.8	0.9	0.7	0.6	1.0	1.0	0.6	0.5	1.4	1.8	1.6	1.1	1.2	1.2	0.9	0.9	0.9	2.3	2.0	1.0	1.0	1.6	1.5	1.0	1.1	0.8	1.3	1.3	0.9	0.4	0.5	0.5	0.3	0.4	0.4	0.8
NES	Nestin	0.9	0.9	1.0	0.7	0.8	0.8	1.0	0.9	1.1	1.1	1.1	1.1	1.7	1.0	1.0	0.9	0.9	0.9	1.0	1.1	1.0	1.1	1.1	1.1	1.1	1.1	1.1	1.2	0.9	0.9	0.9	1.0	0.9	0.9	0.9	1.0	1.2	1.2	1.3	1.1
NEUROD1	NeuroD1	1.2	1.2	1.3	1.1	1.2	1.1	0.8	0.8	0.7	0.9	0.8	0.9	0.6	1.0	1.4	0.8	0.8	0.7	0.0	0.0	0.0	0.0	0.1	0.0	0.0	0.0	0.0	0.0	0.1	0.1	0.0	0.0	0.1	1.7	1.6	1.5	1.1	1.6	1.5	1.0
NEUROG1	Neurogenin1	1.4	1.4	1.4	1.2	1.4	1.0	0.9	1.0	0.2	0.3	1.9	1.8	0.5	0.2	0.2	0.2	0.3	0.2	0.0	0.0	0.0	0.0	0.2	0.1	0.1	0.0	0.0	0.0	0.1	0.1	0.0	0.0	0.1	0.8	0.8	1.0	1.2	1.2	1.4	0.4
NKX2-1	Nkx2.1	1.7	1.8	2.4	0.3	1.2	0.9	0.6	0.7	0.8	1.4	0.4	0.7	0.8	0.5	0.9	1.2	1.1	1.3	1.4	1.1	1.6	0.7	0.2	1.8	1.8	1.9	1.6	1.5	11.5	7.0	0.4	8.7	1.4	0.6	0.3	0.8	1.1	0.2	0.7	1.0
POU5F1	Oct4	0.3	0.2	0.2	6.5	2.4	2.8	0.2	0.2	0.3	0.3	0.2	0.5	0.5	0.2	0.2	0.7	0.8	0.6	0.7	0.7	0.7	0.3	0.3	0.5	0.5	0.5	0.3	0.4	0.5	0.4	0.6	0.5	0.5	0.2	0.2	0.2	0.4	0.5	0.2	0.5
PDE4B	PDE4B	1.6	1.4	1.5	1.1	0.8	0.8	1.1	1.0	0.5	0.5	1.3	1.4	0.8	0.6	0.6	3.4	3.5	3.3	1.7	2.0	1.7	2.7	2.4	1.8	1.9	2.6	2.4	2.2	2.0	1.8	1.9	1.9	1.2	0.6	0.5	0.6	0.7	0.7	0.7	0.4
PICALM	PICALM	1.0	1.0	1.0	0.9	0.9	0.8	0.7	0.7	1.4	1.4	0.7	0.8	1.1	1.2	1.3	1.8	1.6	1.6	1.5	1.6	1.5	1.7	1.4	2.0	1.9	1.6	1.7	2.0	1.9	1.6	1.7	1.7	1.9	1.2	1.3	1.0	1.1	1.3	1.3	1.5
DLG4	PSD-95	0.9	0.9	1.0	0.8	0.8	0.8	0.7	0.7	1.3	1.3	0.8	0.8	1.0	1.6	1.5	0.8	0.7	0.8	0.7	0.7	0.8	1.0	0.8	1.0	1.0	0.6	0.7	0.7	1.3	1.0	0.9	0.8	1.3	0.8	0.8	0.8	0.7	0.7	0.7	0.9
PSEN1	PSEN1	0.9	0.9	0.9	0.9	0.9	0.9	0.8	0.7	1.5	1.5	0.7	0.7	1.1	1.3	1.4	1.7	1.7	1.6	1.6	1.5	1.5	1.4	1.2	1.6	1.6	1.3	1.4	1.6	1.6	1.4	1.4	1.4	1.6	1.1	1.1	1.0	1.0	1.1	1.1	1.9
PSEN2	PSEN2	1.0	1.0	1.0	1.1	1.0	1.0	1.1	1.0	0.8	0.7	1.3	1.5	0.9	0.8	0.7	1.2	1.2	1.2	1.9	1.8	1.8	2.2	1.9	2.0	2.1	2.2	2.2	2.2	1.0	1.0	1.6	1.6	1.6	0.9	1.0	1.0	1.0	1.1	1.1	0.8
PVALB	PVALB	0.7	0.7	0.4	0.9	0.8	0.9	1.0	1.2	1.0	1.0	1.1	1.4	1.1	1.6	1.2	2.2	3.0	2.3	3.6	3.3	3.5	1.6	1.3	1.5	1.5	2.4	2.6	1.5	1.3	1.1	2.6	1.5	0.7	0.7	0.6	0.9	1.3	1.5	1.6	0.7
PAX6	Pax6	0.7	0.7	0.8	0.6	0.6	0.7	0.7	0.7	1.8	1.9	0.7	0.6	1.1	1.5	1.7	0.0	0.0	0.1	0.1	0.1	0.1	0.0	0.1	0.0	0.0	0.0	0.0	0.0	0.0	0.0	0.0	0.0	0.1	0.8	0.8	0.8	0.7	0.9	0.7	1.5
RELN	Reelin	1.2	1.2	1.4	0.5	0.5	0.5	3.0	2.8	0.3	0.3	1.2	1.0	0.3	0.4	0.4	0.6	0.2	0.3	0.4	0.4	0.5	1.8	1.1	4.6	4.7	1.0	1.2	4.1	5.9	4.5	2.5	2.1	3.0	1.1	1.2	1.3	0.5	0.3	0.4	0.8
S100B	S100b	0.6	0.6	0.6	0.4	0.4	0.5	2.2	1.9	0.5	0.5	1.9	2.1	1.1	0.8	0.8	73.9	68.5	57.1	32.1	33.8	34.0	90.5	80.1	42.5	42.6	66.1	66.7	52.6	67.6	63.2	53.1	48.5	44.1	1.0	1.0	1.0	1.6	1.8	2.3	1.3
SORL1	SORLA	0.6	0.6	0.8	1.1	1.5	1.1	0.7	0.7	1.4	1.5	0.6	0.6	1.0	1.3	1.5	0.6	0.6	0.7	0.5	0.6	0.5	0.6	0.6	1.2	1.5	0.7	0.6	1.1	1.4	1.1	0.6	0.6	0.9	0.8	0.7	0.7	1.1	1.4	1.3	1.9
SYP	SYP	1.2	1.1	1.1	0.9	0.9	0.8	0.7	0.7	0.6	0.7	1.3	1.5	0.9	1.3	1.3	1.1	1.2	1.2	1.0	1.0	1.0	2.1	1.8	1.1	1.1	2.1	1.9	1.8	1.6	1.3	1.4	1.5	1.2	0.7	0.7	0.6	0.6	0.7	0.7	0.3
SATB1	Satb1	0.8	0.8	0.8	1.1	1.1	1.1	0.8	0.7	1.3	1.3	0.7	0.7	1.0	1.4	1.4	1.6	1.6	1.6	1.8	1.8	1.8	2.2	1.8	1.8	1.8	1.5	1.6	1.6	1.3	1.1	1.5	1.4	1.6	1.2	1.2	1.2	1.0	1.1	1.2	1.4
SIX3	Six3	1.0	1.0	1.1	0.8	0.7	0.7	0.5	0.4	1.1	0.9	1.5	1.3	1.4	1.3	1.2	0.0	0.0	0.0	0.1	0.1	0.1	0.1	0.1	0.0	0.0	0.0	0.0	0.0	0.0	0.0	0.0	0.0	0.0	0.4	0.4	0.6	0.9	0.8	0.8	0.6
SOX1	Sox1	1.2	1.2	1.3	0.9	1.2	1.0	0.6	0.6	0.9	1.2	0.8	0.8	1.1	0.8	1.2	0.0	0.0	0.0	0.0	0.0	0.0	0.0	0.2	0.0	0.0	0.0	0.0	0.0	0.0	0.0	0.0	0.0	0.0	1.2	1.2	0.9	1.1	1.6	1.6	1.3
SOX2	Sox2	0.7	0.7	0.7	0.9	1.0	0.9	0.7	0.6	1.5	1.5	0.6	0.6	1.6	1.5	1.5	0.1	0.1	0.1	0.2	0.2	0.2	0.1	0.2	0.1	0.1	0.2	0.1	0.1	0.1	0.1	0.2	0.2	0.1	1.2	1.3	1.2	1.3	1.5	1.6	1.2
SYN1	Synapsin	1.0	1.0	1.2	0.9	0.9	0.9	1.0	0.9	0.8	0.9	1.3	1.1	0.8	1.1	1.1	0.4	0.3	0.3	0.5	0.5	0.5	1.1	0.9	0.7	0.8	0.8	0.8	0.9	0.8	0.7	0.7	0.7	0.8	0.8	0.7	0.7	0.5	0.7	0.8	0.4
ADAM17	TACE/ADAM	0.7	0.7	0.7	0.8	0.8	0.8	0.7	0.6	1.8	1.7	0.6	0.7	1.4	1.5	1.5	1.3	1.3	1.2	1.2	1.2	1.1	1.1	0.9	1.3	1.2	1.0	1.0	1.2	1.3	1.1	1.1	1.0	1.3	1.1	1.1	1.1	1.1	1.1	1.1	1.9
MAPT	TAU 3R	1.1	1.1	1.2	0.9	0.9	0.8	0.9	0.9	0.8	0.8	1.2	1.3	0.7	1.2	1.2	1.0	1.1	1.0	0.9	1.2	1.0	1.7	1.7	0.9	0.9	1.6	1.4	1.1	1.1	1.0	1.0	1.1	0.9	0.7	0.7	0.6	0.4	0.4	0.5	0.3
MAPT	TAU 4R	1.1	1.2	1.2	0.4	1.0	0.8	1.1	1.7	0.4	0.5	1.9	2.1	0.5	1.0	0.2	1.6	1.0	0.7	1.5	1.4	1.7	10.7	9.8	3.3	3.9	6.7	7.0	3.1	4.4	3.7	4.4	5.2	4.3	1.5	1.5	1.1	0.7	0.9	0.6	0.6
MAPT	TAU ALL	1.2	1.2	1.4	0.9	0.9	0.8	0.9	0.9	0.7	0.7	1.1	1.1	0.6	1.3	1.3	1.0	1.1	1.1	1.0	1.1	1.1	1.7	1.7	0.8	0.9	1.5	1.4	1.1	1.3	1.1	1.0	1.2	0.9	0.6	0.6	0.6	0.3	0.4	0.4	0.4
TNF	TNF	0.7	0.9	0.6	1.1	1.4	1.3	0.7	1.1	1.2	2.0	0.4	0.5	2.0	0.7	0.5	2.4	1.7	1.3	1.3	1.3	1.5	2.0	1.2	0.7	2.0	2.1	1.6	1.6	2.0	1.4	1.9	1.3	2.4	1.1	0.9	0.9	3.8	1.3	1.5	6.0
TSNAX	TSNAX ex3/4	1.0	1.0	0.9	0.9	0.8	0.8	0.8	0.7	1.5	1.4	0.8	0.9	1.1	1.3	1.2	1.5	1.5	1.4	1.5	1.5	1.3	1.1	1.1	1.5	1.4	1.3	1.4	1.5	1.3	1.2	1.2	1.1	1.5	0.9	1.0	0.8	1.0	1.0	0.9	1.4
TSNAX-DISC	TSNAX-DISC	0.6	0.7	0.8	0.4	0.6	0.6	0.5	0.5	2.6	2.4	0.5	0.5	1.9	1.3	1.0	0.6	0.7	0.6	1.4	1.6	1.3	0.7	0.8	1.1	1.1	0.6	0.9	0.9	1.2	1.0	0.8	0.7	1.7	1.1	1.2	1.3	1.3	1.6	1.1	2.1
TWIST1	TWIST1	0.3	0.3	0.3	0.8	0.6	0.6	0.7	0.5	3.9	3.4	0.9	0.8	1.6	0.1	0.2	7.6	8.4	8.3	18.5	19.0	19.9	10.7	6.5	10.9	9.3	21.4	25.0	19.9	10.0	9.0	14.2	14.7	9.3	2.1	2.2	2.3	7.8	8.6	11.4	0.9
TBR1	Tbr1	1.2	1.1	1.2	0.6	0.7	0.7	1.5	1.6	1.0	0.9	1.3	1.4	0.8	0.5	0.5	0.0	0.0	0.0	0.0	0.0	0.0	0.0	0.0	0.0	0.0	0.0	0.0	0.0	0.0	0.0	0.0	0.0	0.0	1.1	1.1	1.2	0.6	0.6	0.6	0.3
EOMES	Tbr2	2.1	2.2	2.2	1.8	1.3	1.5	0.8	0.8	0.2	0.2	0.6	0.7	0.3	0.2	0.2	0.0	0.0	0.0	0.0	0.0	0.0	0.0	0.0	0.0	0.0	0.0	0.0	0.0	0.0	0.0	0.0	0.0	0.0	0.4	0.4	0.5	0.3	0.3	0.3	0.1
SOX2	Tuj1	0.9	0.9	0.9	0.8	0.7	0.7	0.7	0.7	1.0	1.0	1.4	1.4	1.4	1.3	1.2	0.7	0.7	0.7	0.5	0.6	0.6	1.0	0.9	0.6	0.7	0.9	0.8	1.0	0.5	0.5	0.6	0.7	0.5	0.8	0.8	0.8	1.0	1.2	1.4	0.4
SLC32A1	VGAT	0.8	1.0	1.1	0.8	0.7	0.6	0.3	0.4	0.6	1.0	1.3	1.3	1.0	1.9	2.1	0.2	0.2	0.2	0.2	0.1	0.2	0.1	0.1	0.2	0.1	0.2	0.2	0.2	0.2	0.0	0.0	0.0	0.0	0.5	0.4	0.4	0.3	0.6	0.4	0.4
SLC17A7	VGLUT1	0.4	0.4	0.4	1.0	0.8	1.1	0.9	0.9	2.6	2.2	0.4	0.4	2.0	0.8	0.7	0.8	0.9	0.8	1.0	1.3	1.2	3.0	2.7	1.2	1.2	2.9	2.8	2.2	1.3	0.9	1.6	1.8	0.6	2.1	1.8	2.0	1.6	1.9	1.7	0.6
SLC17A6	VGLUT2	1.4	1.3	1.4	1.1	1.0	1.1	1.0	1.1	0.7	0.6	1.1	1.1	0.6	0.8	0.6	1.8	2.0	2.0	1.0	1.1	1.1	1.1	1.0	0.3	0.3	0.7	0.7	0.4	0.9	0.8	0.6	0.5	0.5	1.0	1.0	1.1	0.6	0.6	0.7	0.3
VIM	Vimentin	0.7	0.7	0.7	0.8	0.8	0.9	0.7	0.7	1.4	1.5	0.9	1.0	1.6	1.2	1.3	1.1	1.0	1.0	1.4	1.5	1.5	1.5	1.2	1.6	1.6	1.7	1.8	1.9	1.8	1.4	1.7	1.6	1.6	1.3	1.3	1.1	1.7	2.1	2.2	1.7
WNT3A	WNT3A	0.8	0.9	1.1	0.5	0.3	0.5	2.1	2.1	0.8	0.7	1.7	1.5	1.6	0.4	0.2	2.7	3.0	2.8	6.7	6.1	7.7	1.6	1.4	3.0	2.6	4.3	4.3	3.6	2.2	1.6	2.9	2.3	2.3	1.2	1.3	1.5	3.1	2.9	3.0	0.8
OLFM1	Y domain	1.1	1.1	1.1	0.9	0.9	0.8	1.0	1.1	0.5	0.6	1.2	1.4	0.6	1.3	1.4	1.3	1.4	1.5	0.7	0.8	0.7	0.7	0.7	0.4	0.5	0.7	0.7	0.6	0.8	0.7	0.5	0.6	0.5	1.1	1.1	1.0	0.7	0.9	0.9	0.4
OLFM1	Z Domain	0.9	0.9	0.9	0.9	0.9	0.9	1.0	1.0	0.7	0.8	1.0	1.1	0.8	1.6	1.7	0.7	0.7	0.8	0.5	0.6	0.5	0.5	0.5	0.3	0.4	0.5	0.5	0.4	0.6	0.5	0.4	0.4	0.4	1.1	1.1	1.1	0.7	0.8	0.9	0.4
ZIC2	ZIC2	1.2	1.2	1.3	0.8	1.0	0.8	1.1	1.1	1.2	1.2	0.8	0.8	0.9	0.7	0.9	0.5	0.5	0.6	1.0	1.1	1.0	0.5	0.4	0.2	0.2	0.6	0.6	0.2	0.3	0.3	0.7	0.7	0.1	1.3	1.3	1.2	1.1	1.3	1.3	1.5
ZIC3	ZIC3	1.4	1.2	1.3	0.8	0.9	0.6	1.5	1.4	0.7	0.6	1.2	1.3	0.7	0.6	0.7	0.3	0.3	0.3	0.8	0.8	0.8	0.4	0.4	0.1	0.1	0.7	0.6	0.1	0.2	0.2	0.5	0.4	0.1	1.7	1.7	1.7	1.6	1.8	1.8	1.4
GPHN	gephyrin	1.2	1.2	1.4	1.0	1.4	1.0	0.8	0.9	0.8	0.8	0.9	1.1	0.9	0.7	0.8	1.5	1.5	1.4	0.8	0.9	0.8	1.0	0.9	1.4	1.5	1.0	1.1	1.5	1.6	1.6	0.9	1.0	1.3	1.0	1.0	0.8	0.9	1.3	1.3	1.0
GLUL	glutamine syr	1.1	1.1	1.1	0.9	0.9	0.9	0.7	0.6	1.2	1.3	0.6	0.7	1.0	1.4	1.5	2.3	2.1	1.9	1.1	1.0	1.0	0.7	0.6	1.0	1.0	0.7	0.7	0.9	1.5	1.2	0.7	0.7	1.1	1.1	1.1	1.0	1.0	1.2	1.2	1.8
NANOG	nanog	0.5	0.4	0.3	7.0	2.4	3.0	0.3	0.2	0.1	0.0	0.1	0.3	0.3	0.0	0.0	0.6	0.4	0.7	0.3	0.3	0.3	0.1	0.2	0.3	0.4	0.2	0.1	0.2	0.2	0.2	0.3	0.3	0.2	0.1	0.1	0.1	0.3	0.2	0.0	0.1
TP53	p53	1.1	1.1	1.2	0.8	0.9	0.9	0.8	0.9	1.0	1.4	0.8	0.9	0.9	0.9	1.4	1.6	1.5	1.3	0.8	0.9	0.8	0.8	0.8	1.3	1.5	1.0	1.0	1.4	1.7	1.6	0.8	0.9	1.1	1.4	1.5	1.1	1.0	1.5	1.6	1.6
TP73	p73	0.6	0.4	0.5	0.4	0.5	0.5	2.7	2.7	1.1	1.0	1.5	1.7	0.9	0.2	0.2	0.5	0.8	0.6	2.0	1.8	1.5	0.4	0.3	0.6	0.7	0.5	0.6	0.5	0.4	0.4	0.3	0.6	0.5	3.5	3.6	4.1	2.0	2.2	2.5	1.1
